# An Injectable and Self‐Reinforced Piezoelectric Hydrogel for Irregular Bone Defects Regeneration: Dynamic Epigenetic Regulation Approach

**DOI:** 10.1002/advs.77911

**Published:** 2026-10-06

**Authors:** Yu‐Kai Huang, Juan Li, Miao‐Miao Yang, Chao Jia, Quan‐Xin Gong, Guang‐Cheng Yuan, Dong Zhou, Wen‐Hui Shen, Zeng‐Gan Chen, Zhi‐Rui Dong, Jin‐Wang Huang, Jie Peng, Di‐Han Su, Ming‐Dong Zhao, Jian Dong, Li‐Bo Jiang

**Affiliations:** ^1^ Department of Orthopaedic Surgery Zhongshan Hospital Fudan University Shanghai People's Republic of China; ^2^ Department of Orthopaedics Jinshan Hospital Fudan University Shanghai People's Republic of China; ^3^ Department of Nursing Zhongshan Hospital Fudan University Shanghai People's Republic of China; ^4^ School of Materials and Chemistry University of Shanghai for Science and Technology Shanghai People's Republic of China; ^5^ Frontiers Science Center for Materiobiology and Dynamic Chemistry School of Materials Science and Engineering Key Laboratory for Ultrafine Materials of Ministry of Education Research Center for Biomedical Materials of Ministry of Education East China University of Science and Technology Shanghai People's Republic of China; ^6^ Department of Orthopedic Surgery Zhongshan Hospital (Xiamen) Fudan University Fujian People's Republic of China; ^7^ Xiamen Osteoporosis Specialized Prevention and Treatment Center Xiamen People's Republic of China; ^8^ Clinical Medical College of Xiamen Medical College Fujian People's Republic of China

**Keywords:** acetylation, osteogenic activity, piezoelectric hydrogel, self‐reinforcing, silk fibroin

## Abstract

Irregular bone defects demand biomaterials that can be delivered into complex shapes while offering gradually developing mechanical strength and bioactive electrical stimulation‐two critical properties rarely obtained simultaneously. Here, we develop an injectable, polymer‐induced self‐reinforcing and piezoelectric hydrogel engineered from regenerated silk fibroin, tyramine‐modified CMC, and MXene‐cellulose nanofiber nanosheets through enzymatically dynamic Cross‐linking. This material is designed to overcome two major limitations of current injectable hydrogels: inadequate mechanical maturation and unstable bioelectrical output. The dynamic dual‐network structure undergoes gradual β‐sheet alignment and nanointerfacial ordering, which increases the compressive modulus from ∼0.08 to ∼0.8 MPa and elevates piezoelectric output from 3 to 60 mV over 5 days, establishing a mechanically stable and electrically active microenvironment within the defect. The hydrogel‐derived electrical signals induce Ca^2+^ influx and activate the CaMKII/CREB/P300 axis, enhancing H3K9 acetylation and reinitiating osteogenic transcription, with PTH1R emerging as a key downstream effector. This hydrogel promotes osteogenic differentiation, mineralization, and angiogenesis in vitro and accelerates new bone formation while restoring trabecular architecture in critical‐sized defects in vivo. Overall, this mechanoadaptive hydrogel provides sustained piezoelectric stimulation while functioning as an epigenetic activator to reprogram the osteogenic microenvironment, offering a promising strategy for irregular bone defect repair.

## Graphical abstract

An injectable and self‐reinforcing RSF‐based piezoelectric hydrogel conforms to irregular bone defects and progressively strengthens in situ, overcoming the trade‐off between injectability and mechanical stability. Mechanical loading generates bioelectrical cues that promote P300‐mediated H3K9 acetylation and upregulate PTH1R expression, thereby enhancing osteogenesis and angiogenesis. This adaptive mechanoelectrical–epigenetic platform enables robust regeneration of complex bone defects.

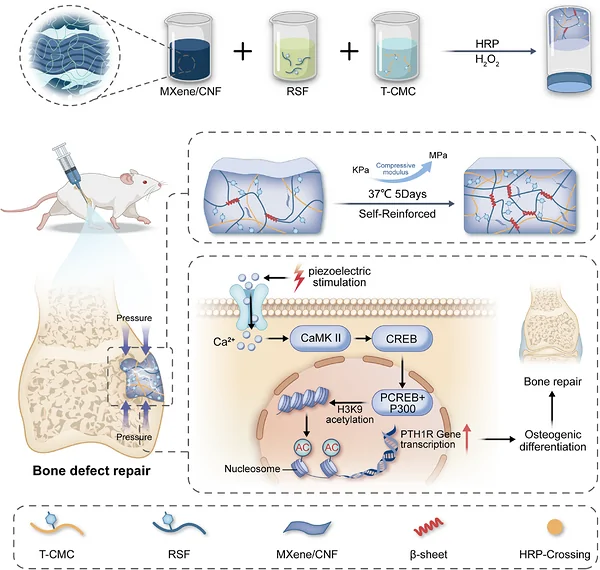

## 1 Introduction

Bone defects caused by trauma, infection, or tumor resection remain a common clinical problem and often result in significant functional impairment and disability [1]. Although autologous bone grafting is still considered the clinical “gold standard” [2, 3], complications such as donor site morbidity, limited graft quantity, and surgical risks emphasize the need for alternative biomaterials [4, 5]. Ideal candidates for irregular bone defect repair should combine injectability for minimally invasive placement with adequate mechanical strength throughout the regeneration period [6, 7]. However, conventional injectable hydrogels, despite their ability to adapt to irregular shapes, inherently lack load‐bearing strength and often require external triggers—such as photo‐crosslinking or chemical reinforcement—to achieve proper mechanical performance [8, 9, 10]. These external strengthening steps may improve stiffness but increase procedural complexity and can limit clinical usefulness. Thus, developing hydrogels that can autonomously self‐reinforce after injection is a more practical and favorable direction.

Bone is naturally piezoelectric, with collagen and hydroxyapatite converting mechanical stimuli into electrical signals that regulate osteogenesis [11, 12]. Restoring the biophysical microenvironment of bone defects requires both mechanical stability and appropriate electrical cues. Disruption of this endogenous electrical environment impairs regeneration [13]. While exogenous electrical stimulation enhances osteogenic differentiation of BMSCs, its clinical use is restricted by invasiveness, infection risk, and reliance on bulky external devices [14, 15, 16]. Piezoelectric biomaterials capable of generating localized electrical signals under physiological loading have therefore attracted increasing interest. Piezoelectric hydrogels can convert mechanical deformation into bioelectric cues that promote osteogenesis and partially restore the native electrical environment [17, 18]. Yet many existing systems still struggle with limited mechanical robustness, unstable piezoelectric output, or poor injectability, highlighting the need for materials that integrate these functions in a clinically feasible way.

To address these requirements, researchers have increasingly focused on natural polymer‐based hydrogels due to their inherent biocompatibility and their ability to form reinforcing molecular interactions. Regenerated silk fibroin (RSF) is particularly appealing for minimally invasive bone repair because of its injectability, mild gelation process, and tunable degradation profile [19, 20]. Nevertheless, its native mechanical strength remains insufficient for load‐bearing applications [21, 22, 23]. To enhance the mechanical robustness of RSF hydrogels, various complementary components have been incorporated to strengthen intermolecular interactions and network structure. For instance, additives such as chitosan, alginate, or hyaluronic acid can increase hydrogen bonding and chain entanglement, while nano‐hydroxyapatite, bioactive glass, or silica nanostructures offer additional mechanical support and osteoconductive cues. However, although these formulations demonstrate that RSF can indeed be reinforced, many individual systems remain difficult to translate into injectable platforms, often losing flowability at higher concentrations or showing limited post‐injection mechanical maturation [24, 25]. Among cellulose‐derived polysaccharides, carboxymethyl cellulose (CMC) is notable because it has consistently been shown to enhance the mechanical and rheological stability of various injectable hydrogels—including thermosensitive F127 gels, HA‐based in situ forming gels, and γ‐PGA adaptive networks—while maintaining flowability and cytocompatibility. These observations highlight CMC as a flexible co‐matrix capable of reinforcing soft hydrogel systems, suggesting promising compatibility with RSF‐based injectable matrices [26, 27, 28].

Meanwhile, the need to restore electrical signaling has driven the use of electroactive materials [29, 30]. Current piezoelectric biomaterials for bone repair primarily involve inorganic piezoelectric ceramics (e.g., BaTiO_3_, ZnO) and increasingly explored organic/inorganic hybrid scaffolds. However, despite their strong electrical output, these nanoparticles suffer from inherent rigidity, limited injectability, and poor biodegradability, making them unsuitable for irregular or minimally invasive defect sites. Hybrid hydrogels that incorporate piezoelectric nanoparticles improve flexibility but often face issues such as particle aggregation, unstable output under physiological hydration, and weak electromechanical coupling within the soft matrix [31, 32]. Recently, two‐dimensional MXenes have emerged as promising electroactive materials. Their high conductivity, excellent hydrophilicity, and plentiful surface terminations support strong interactions with polymer networks and facilitate stress‐induced dipole alignment, making them well‐suited for developing injectable hydrogels capable of providing stable and physiologically relevant electroactive cues [33, 34, 35]. Notably, recent evidence indicates that such electromechanical cues influence not only cell behavior but also the underlying epigenetic landscape [36, 37]. Among these mechanisms, histone acetylation (e.g., H3K9ac) represents a dynamic and reversible modification closely linked with transcriptional activation, allowing cells to respond rapidly to biophysical input [38, 39, 40]. While exogenous electrical stimulation has been shown to alter epigenetic states in several cell types, how stem cells perceive piezoelectric signals generated directly from biomaterials remains largely unresolved [29]. Understanding this mechanism is key for clarifying how electromechanically active scaffolds influence stem‐cell fate and for designing next‐generation electroactive biomaterials.

Accordingly, a new RSF‐based hydrogel was developed in which a tyramine‐modified CMC network enables autonomous self‐reinforcement after injection, while an MXene‐CNF‐based electroactive component enhances the effective piezoelectric/electromechanical responsiveness of the composite hydrogel (Figure 1). This integrated system combines mechanical durability with electromechanical and epigenetic regulation, promoting osteogenesis and angiogenesis in vitro and accelerating bone regeneration in vivo.

**FIGURE 1:**
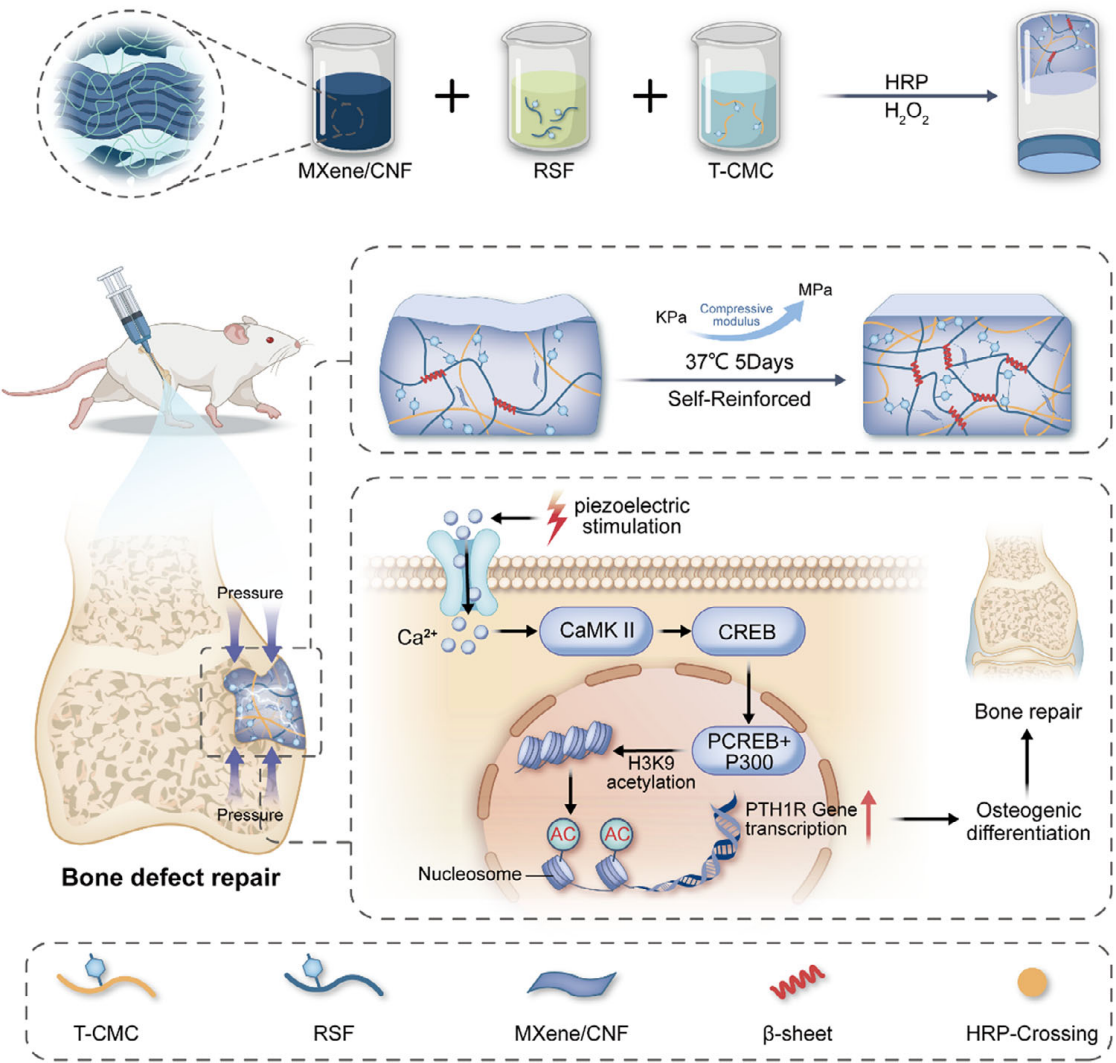
Schematic illustration showing the synthesis of the piezoelectric hydrogel and the proposed mechanism by which mechanical‐electrical conversion induces Ca^2+^ influx and histone H3K9 acetylation, thereby activating PTH1R‐mediated downstream signaling to enhance osteogenic differentiation and bone regeneration.

## 2 Results

### 2.1 Characterization Of CNF‐Modified MXene and Its Electroactive Properties

To clarify how cellulose nanofiber (CNF) modification influences the structure and physicochemical behavior of MXene, a detailed characterization was performed. In the FTIR spectra (Figure 2A), pristine MXene displayed bands at ∼1632 and 644 cm^−1^, corresponding to the bending vibration of adsorbed water/surface hydroxyl groups and Ti─O stretching, respectively. In contrast, CNF showed strong absorption peaks at 3342 cm^−1^ (O─H stretching), 2861 cm^−1^ (C─H stretching), and 1057 cm^−1^ (C─O─C stretching of glycosidic linkages), representing the typical cellulose backbone. The MXene–CNF composite retained these CNF‐associated peaks, while the O─H and C─O─C bands became broader and slightly shifted, suggesting hydrogen‐bond‐mediated interfacial interactions between CNF and the MXene surface terminations (─OH, ─O, ─F) [41, 42].These spectral changes indicate strong hydrogen‐bond‐driven interfacial coupling between MXene and CNF. To further verify this interfacial modification, high‐resolution XPS spectra of the O 1s region were examined for MXene and MXene–CNF (Figure 2B,C). Pristine MXene exhibited three major deconvoluted peaks at 532.61 eV (C─Ti─OH_x_), 530.10 eV (Ti─C─O_x_), and 528.75 eV (TiO_2_), corresponding to surface hydroxyl, mixed oxide, and oxidized titanium states. After CNF grafting, the overall O 1s profile shifted toward higher binding energies, accompanied by a new component at 533.87 eV attributed to C─O bonds from the CNF polysaccharide backbone [43]. In addition, the relative intensity of oxygen‐rich species (C─Ti─OH_x_ and C─O) increased, whereas that of TiO_2_ slightly decreased, implying partial surface coverage of MXene by CNF chains that helps inhibit further oxidation. These changes collectively indicate that CNF introduces hydroxyl‐ and ether‐rich interfacial environments around MXene sheets, improving hydrophilicity and interfacial polarization capability [44]. The presence of C─O and C─Ti─OH_x_ bonds also implies strong hydrogen‐bonding between CNF and MXene surface terminations, which may promote charge redistribution and stabilize the electronic environment for enhanced dielectric behavior [45, 46]. SEM and TEM images (Figure 2D,E) showed that MXene–CNF formed an intertwined structure composed of flexible CNF nanofibers interlaced with layered MXene sheets, providing structural integrity and abundant interfacial contact. To determine whether CNF modification enhances the electrical response of MXene within composites, a model system was prepared using poly(vinyl alcohol) (PVA) as a neutral matrix owing to its good dielectric stability and processability [47, 48, 49]. The dielectric properties of these PVA composites were then analyzed (Figure 2F and Figure S1). Compared with pure PVA and PVA/MXene, the PVA/MXene–CNF composite showed a significantly higher dielectric constant and lower dielectric loss across the tested frequency range. This improvement likely arises from enhanced interfacial polarization introduced by CNF, whose abundant hydroxyl and oxygen‐containing groups promote effective dipole alignment and reduce charge carrier migration. Thus, CNF‐modified MXene exhibits a more stable dielectric response with lower energy dissipation, confirming its improved dielectric and electroactive functionality [45]. EDS elemental mapping (Figure 2G) further confirmed the uniform distribution of Ti, C, F, and O, indicating homogeneous incorporation of MXene and CNF within the composite matrix, which supports the observed enhancement in electrical properties. To further evaluate the electromechanical contribution of the MXene‐CNF hybrid structure, PFM characterization was performed on MXene and MXene‐CNF alone. Compared with MXene, MXene‐CNF exhibited stronger PFM amplitude and phase responses, as shown by both PFM mapping and bias‐dependent amplitude–voltage/phase–voltage measurements (Figure S7). These results suggest that CNF hybridization enhances the local electromechanical response of the MXene‐based hybrid structure, likely by strengthening hydrogen‐bond‐mediated interfacial interactions, promoting interfacial polarization and charge redistribution between MXene nanosheets and the surrounding matrix.

**FIGURE 2:**
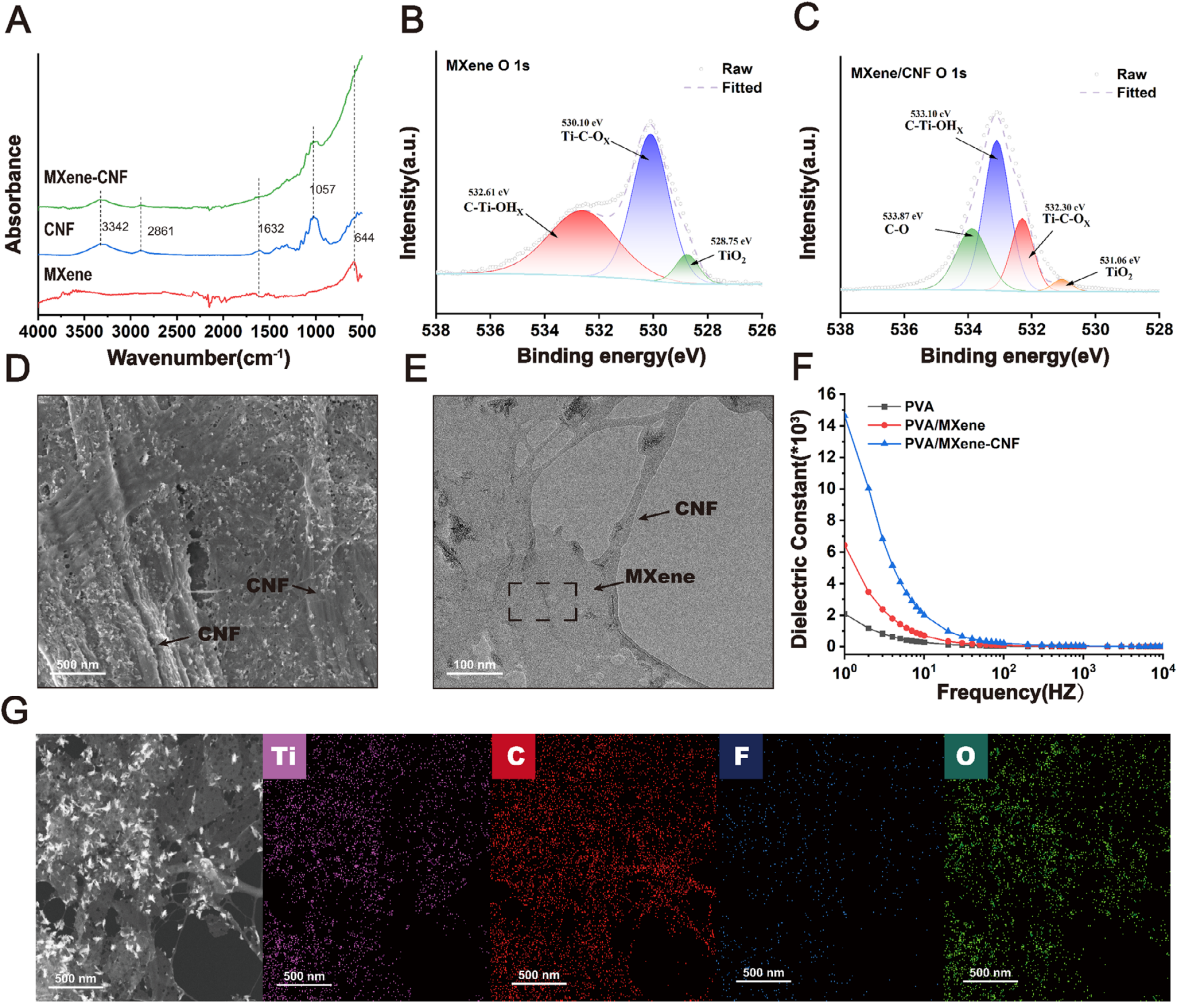
Characterization and electroactive properties of CNF‐modified MXene composites. (A) FTIR spectra of CNF, MXene, and MXene‐CNF composites. (B, C) XPS spectra of the O 1s region for MXene and MXene‐CNF. (D) SEM image of MXene‐CNF. (E) TEM image of MXene‐CNF. (F) Frequency‐dependent dielectric constant of PVA, PVA/MXene, and PVA/MXene‐CNF composites. (G) EDS elemental mapping of Ti, C, F, and O.

### 2.2 Enzymatic Crosslinking‐Induced Structural Evolution and Mechanistic Origin of Self‐Reinforcement

As shown in Figure 3A, RSF was combined with a tyramine‐modified CMC (T‐CMC) network and a uniform distribution of MXene–CNF nanosheets. The HRP/H_2_O_2_ enzymatic system was used to trigger phenolic crosslinking, producing a covalently reinforced network. The injectable precursor then gradually self‐strengthened into a robust hydrogel. Successful synthesis of T‐CMC, a key component enabling enzymatic crosslinking and secondary network stabilization, was confirmed by ^1^H NMR spectroscopy (Figure 3B), where characteristic aromatic proton peaks of tyramine were observed [50]. As shown in Figure 3C,G, increasing the T‐CMC concentration significantly shortened the gelation time—from ∼300 to ∼100 s—because the introduction of tyramine‐derived phenolic hydroxyl groups added reactive sites, accelerating dityramine bond formation and promoting faster network nucleation [51]. The incorporation of MXene–CNF further improved the gelation process: hydroxyl groups on MXene provided extra hydrogen‐bonding sites that strengthened interfacial interactions with RSF, while CNF chains formed dense hydrogen‐bond bridges that enhanced polymer‐segment interactions [33, 52, 53]. This synergistic combination promoted tighter molecular packing, faster network formation, and a more uniform sol–gel transition. Time‐sweep rheological analysis (Figure 3F) supported these observations: the storage (G′) and loss (G″) moduli of RSF, T‐CMC/RSF (TCR), and 0.4% MXene‐CNF/T‐CMC/RSF (0.4% MC@TCR) samples all showed a sharp increase upon thermal induction, indicating the sol–gel transition. Notably, G′ and G″ in MXene‐CNF‐containing hydrogels increased more rapidly and reached a plateau earlier than in RSF or TCR, reflecting accelerated crosslinking kinetics and faster network stiffening. The macroscopic appearance of RSF, TCR, and 0.2%, 0.4%, and 0.8% MC@TCR hydrogels (Figure 3D and Figure S2) demonstrated rapid sol–gel transition and excellent injectability, enabling uniform filling of irregular defects in a porcine femur model (Figure 3E). SEM and Cryo‐SEM (Figure 3H and Figures S3, S6) revealed that T‐CMC introduction produced a denser and more interconnected porous network, whereas MXene‐CNF further compacted the pore walls into a hierarchically reinforced microarchitecture. EDS mapping (Figure 3I and Figures S4,S5) confirmed uniform distribution of Ti, C, N, and O, verifying homogeneous dispersion of MXene‐CNF within the hydrogel matrix.

**FIGURE 3:**
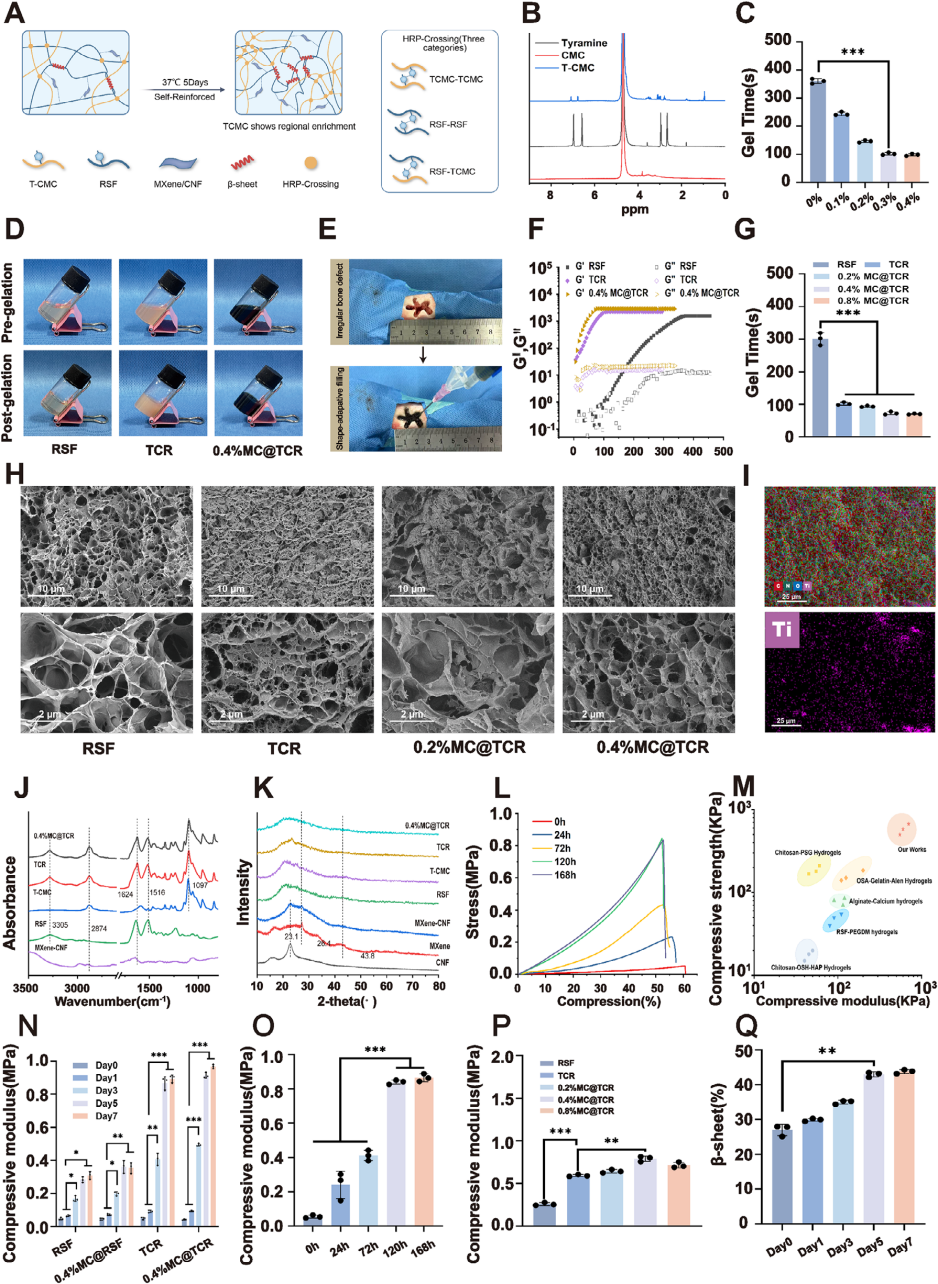
Characterization and self‐reinforcement of RSF/MXene‐CNF/T‐CMC hydrogels. (A) Schematic illustration of enzymatic crosslinking and thermal self‐reinforcement mechanism. (B) ^1^H NMR confirming successful synthesis of T‐CMC. (C) Gelation time of hydrogels with different concentrations of T‐CMC. (D) Macroscopic appearance of RSF, TCR, and 0.4% MC@TCR hydrogels. (E) Injectable shape‐adaptive 0.4% MC@TCR hydrogel filling an irregular defect in porcine femur. (F) Time‐sweep rheology curves showing sol–gel transition. (G) Gelation time of RSF, TCR, and MC@TCR hydrogels with different MXene‐CNF concentrations. (H) SEM images of hydrogel cross‐sections. (I) SEM–EDS elemental mapping. (J) FTIR spectra. (K) XRD patterns. (L, O) Compression‐stress curves and modulus after different reinforcement times. (M) Ashby plot comparison. (N) Comparison of compression modulus after reinforcement in vivo. (P) Modulus of hydrogels with varied MXene‐CNF concentrations. (Q) β‐sheet comparison by amide III deconvolution. ***p < 0.001, **p < 0.01, *p < 0.05, *n* = 3.

To elucidate the molecular interactions and secondary‐structure evolution, FTIR and XRD analyses were conducted (Figure 3J,K). In the FTIR spectra (Figure 3J), RSF displayed characteristic amide I and II bands at 1624 and 1516 cm^−1^ and an N─H stretch at 3305 cm^−1^. After incorporating T‐CMC and MXene‐CNF, the amide peaks became sharper and slightly shifted, indicating enhanced hydrogen‐bond interactions and conformational rearrangement of RSF chains. The new absorption near 1097 cm^−1^ originated from C─O─C stretching of T‐CMC/CNF backbones, confirming successful compositional integration. Notably, the 0.4% MC@TCR hydrogel exhibited a strengthened β‐sheet‐associated amide I band, suggesting that MXene‐CNF promotes molecular alignment and secondary‐structure stabilization through interfacial confinement. XRD patterns (Figure 3K) further supported these observations: MXene showed a (002) reflection near 6.4°, whereas CNF displayed a broad amorphous halo. MXene‐CNF exhibited new reflections at 23.1° and 28.4°, indicating interlayer rearrangement induced by CNF modification. RSF and T‐CMC presented β‐sheet diffraction around 20.4°, and the 0.4% MC@TCR hydrogel showed the highest β‐sheet‐related intensity, demonstrating increased crystallinity and hierarchical structural ordering. Semi‐quantitative deconvolution of the amide III band (Figure 3Q) confirmed a continuous rise in β‐sheet content during thermal incubation, indicating time‐dependent structural reorganization. These molecular adjustments produced a marked improvement in mechanical performance. The compressive modulus of the optimized 0.4% MC@TCR hydrogel rose from approximately 0.08 MPa at Day 0 to about 0.85 MPa by Day 5 (Figure 3L,O), reflecting nearly a ten‐fold rise, while RSF and TCR showed only limited increases. A comparable self‐strengthening behavior was also noted in vivo (Figure 3N), and evaluation of various MXene–CNF concentrations (Figure 3P) indicated that the 0.4% composition provided the most suitable balance between stiffness and toughness. Importantly, the Ashby plot comparison (Figure 3M) shows that this hydrogel occupies a distinctly superior region of the mechanical‐injectability landscape relative to other self‐reinforcing, biomacromolecule‐modified injectable hydrogels, highlighting a level of mechanical robustness rarely achieved in this class of materials [54, 55, 56, 57, 58]. Stress relaxation and tensile tests were further performed to evaluate the mechanical properties of the hydrogels. The 0.4% MC@TCR hydrogel retained the highest normalized stress after 600 s under sustained compressive strain, indicating improved viscoelastic stability. In addition, tensile testing showed that 0.4% MC@TCR exhibited the highest tensile stress while maintaining acceptable elongation at break, suggesting enhanced mechanical robustness without excessive brittleness (Figure S8). To further assess the structural stability of the hydrogels, in vitro degradation and swelling assays were performed (Figure S9A,B). All hydrogels showed a gradual decrease in remaining weight over 28 days, while the 0.4% MC@TCR hydrogel retained more than 90% of its original dry weight, indicating a relatively slow degradation profile. The swelling ratio reached approximately 160%–180% within 7 days, suggesting moderate water absorption without excessive expansion. In addition, implanted 0.4% MC@TCR hydrogels harvested at 0, 14, and 28 days retained compressive mechanical properties in vivo, with the highest mechanical performance observed at 14 days and sustained strength at 28 days (Figure S9C). These results indicate that the self‐reinforced hydrogel maintains suitable structural integrity during the early bone repair period.

Mechanistically (Figure 3A), during incubation, the phenolic groups of T‐CMC continue to participate in dynamic enzymatic crosslinking and redistribute within the network, generating localized molecular confinement that restricts RSF chain mobility [59]. This confined environment encourages chain rearrangement and drives the gradual transition of RSF from random coils to aligned β‐sheet structures. As incubation proceeds, these β‐sheets stack into larger lamellar domains, increasing crystallinity and physical crosslink density [60, 61, 62]. Concurrently, MXene–CNF nanosheets function as nanoscale reinforcers and alignment templates, offering abundant hydrogen‐bonding sites and guiding the orientation of β‐sheet domains along their surfaces [63]. The combined effect of T‐CMC–driven confinement and MXene–CNF templating produces a gradually ordered and densely packed network, accounting for the time‐dependent increase in modulus and toughness and providing the structural foundation for the improved piezoelectric and osteogenic responses.

### 2.3 Effective Piezoelectric Performance and Bioelectrical Relevance of the Hydrogel System

Under cyclic compression at 0.1 MPa (Figure 4A,B and Figure S10), the TCR hydrogel generated only ∼10 mV and ∼12 nA, reflecting weak dipole polarization and limited electromechanical coupling. But incorporating MXene–CNF produced a clear, concentration‐dependent enhancement in piezoelectric output. The interplay between T‐CMC–mediated confinement and MXene–CNF templating yields a progressively aligned and compact network, contributing to the time‐dependent enhancement of modulus and toughness and forming the structural basis for the elevated piezoelectric and osteogenic performance.

**FIGURE 4:**
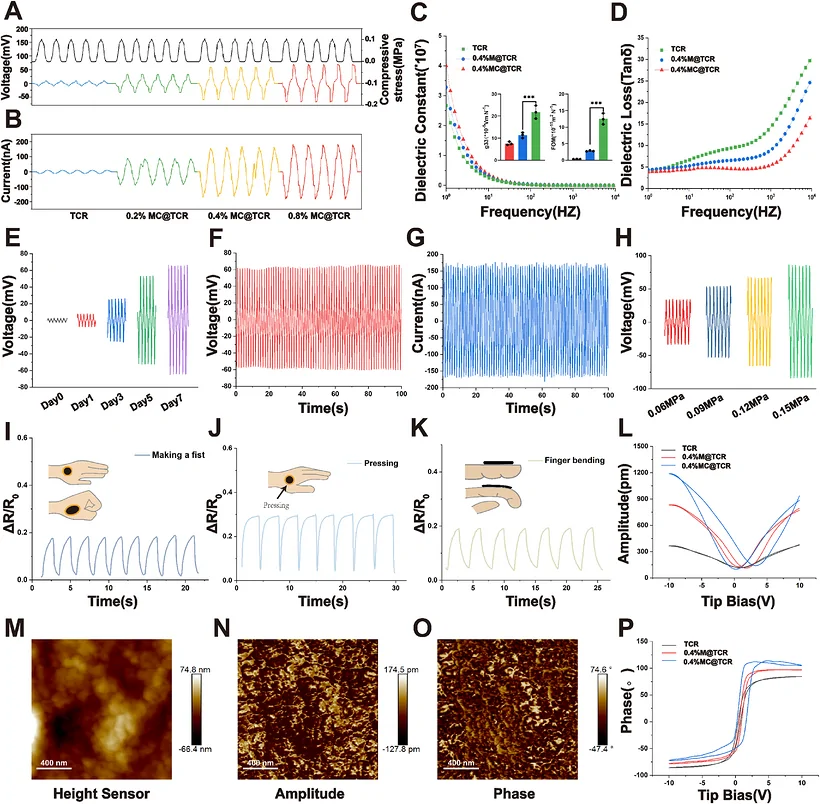
Effective piezoelectric performance and sensing properties of hydrogels. (A, B) Output voltage and current of TCR and MC@TCR hydrogels with different MXene‐CNF concentrations (0.2%, 0.4%, 0.8%, w/v) under 0.1 MPa cyclic compression, showing concentration‐dependent enhancement. (C) Frequency‐dependent dielectric constant of TCR, 0.4% MXene /T‐CMC/RSF (0.4% M@TCR), and 0.4% MC@TCR composites. (D) Frequency‐dependent dielectric loss of TCR, 0.4% M@TCR, and 0.4% MC@TCR composites. (E) Voltage evolution of 0.4% MC@TCR hydrogel at 0.1 MPa over different incubation times (0, 1, 3, 5, and 7 days), reflecting self‐reinforcement. (F, G) Voltage and current outputs of 0.4% MC@TCR hydrogel under continuous compression for 100 s at 0.1 MPa. (H) Pressure‐dependent voltage outputs of 0.4% MC@TCR hydrogel at applied stresses of 0.06, 0.09, 0.12, and 0.15 MPa, confirming load‐responsive behavior. (I–K) Representative electrical signals recorded by sandwich‐structured hydrogel sensors (copper foil/hydrogel/copper foil, PI encapsulated) during fist clenching, hydrogel squeezing, and finger bending. (L) PFM amplitude mapping of TCR, 0.4% M@TCR, and 0.4% MC@TCR hydrogels. (M–O) PFM amplitude images highlighting enhanced nanoscale piezoresponse in 0.4% MC@TCR hydrogels. (P) PFM phase mapping of TCR, 0.4% M@TCR, and 0.4% MC@TCR hydrogels. ***p < 0.001, **p < 0.01, *p < 0.05, *n* = 3.

Dielectric and piezoelectric coefficient analysis (Figure 4C,D and Figure S11) showed that the piezoelectric coefficient peaked at a MXene:CNF ratio of 20:1. Meanwhile, the 0.4% MC@TCR hydrogel exhibited the highest dielectric constant, a g33 value of 22.6 mV·m/N, and a markedly increased figure of merit (FOM) relative to TCR and 0.4%M@TCR, indicating enhanced electromechanical conversion efficiency. The high g_33_ reflects strong voltage generation under mechanical stress, while the increased FOM demonstrates excellent suitability for energy transduction in biological microenvironments [64, 65]. Dielectric loss remained low, supporting stable charge retention necessary for sustained cellular stimulation. Notably, the measured value confirms that the RSF/T‐CMC/MXene‐CNF hydrogel possesses an effective piezoelectric response as an integrated composite system. However, considering that Ti_3_C_2_T_x_ MXene is not a classical piezoelectric crystal, the observed response should not be attributed solely to the intrinsic piezoelectricity of MXene. Instead, the effective piezoelectric response of the composite hydrogel is more reasonably attributed to interface‐mediated electromechanical coupling within the composite network.

The temporal and loading‐dependent features of the piezoelectric response further illustrate the self‐reinforcing nature of the system. For 0.4% MC@TCR, output voltage increased from 3 mV at day 0 to 60 mV by day 7 under the same 0.1 MPa loading, correlating with progressive β‐sheet formation and dipole alignment (Figure 4E). This confirms that structural maturation directly contributes to incremental piezoelectric enhancement. Under continuous cyclic compression for 100 s (Figure 4F,G), the signal remained stable without noticeable decay, demonstrating excellent electromechanical durability. The output amplitude also scaled nearly linearly with applied pressure from 0.06 to 0.15 MPa (Figure 4H), indicating load‐responsive effective piezoelectric/electromechanical behavior rather than random electrical fluctuation. To evaluate whether the hydrogel retained piezoelectric functionality after implantation, 0.4% MC@TCR hydrogels were harvested at 0, 14, and 28 days for voltage output measurement (Figure S9D). The piezoelectric output increased at 14 days and remained detectable at 28 days, indicating that the hydrogel maintained effective piezoelectric activity during the early implantation period.

When integrated into a sandwich‐type device (Figure 4I–K), the MC@TCR hydrogel could distinguish different biomechanical events—including fist clenching, squeezing, and finger bending—producing distinct and reproducible electrical waveforms. This reflects high sensitivity to subtle differences in strain pattern and amplitude. At the nanoscale, Piezoresponse Force Microscopy (Figure 4L–P) showed that MC@TCR hydrogels exhibited the strongest PFM amplitude and a phase contrast approaching 180°, consistent with well‐aligned piezoelectric domains and robust polarization switching, in agreement with the macroscopic voltage–current outputs.

Importantly, this self‐generated electrical output is biologically relevant, as the magnitude and stability of the piezoelectric signal fall within the range known to activate calcium signaling and support osteogenic differentiation [66, 67, 68]. Thus, the structural‐electrical coupling established in the material provides the mechanistic basis for the biological functions explored in the following section.

### 2.4 Biocompatibility and Piezoelectric Stimulation‐Induced Cellular Activation

The interaction between T‐CMC–induced confinement and MXene–CNF templating facilitates the development of a more ordered and tightly consolidated network, which supports the gradual increase in modulus and toughness and underpins the improved piezoelectric and osteogenic outcomes. However, by day 3, a concentration‐dependent decline in cell viability became apparent, especially in the 0.8% group, indicating that higher MXene‐CNF content may exert adverse effects on BMSC proliferation over time (Figure 5A). Considering both biocompatibility and piezoelectric performance, 0.4% MXene‐CNF was selected as the optimal formulation for subsequent studies.

**FIGURE 5:**
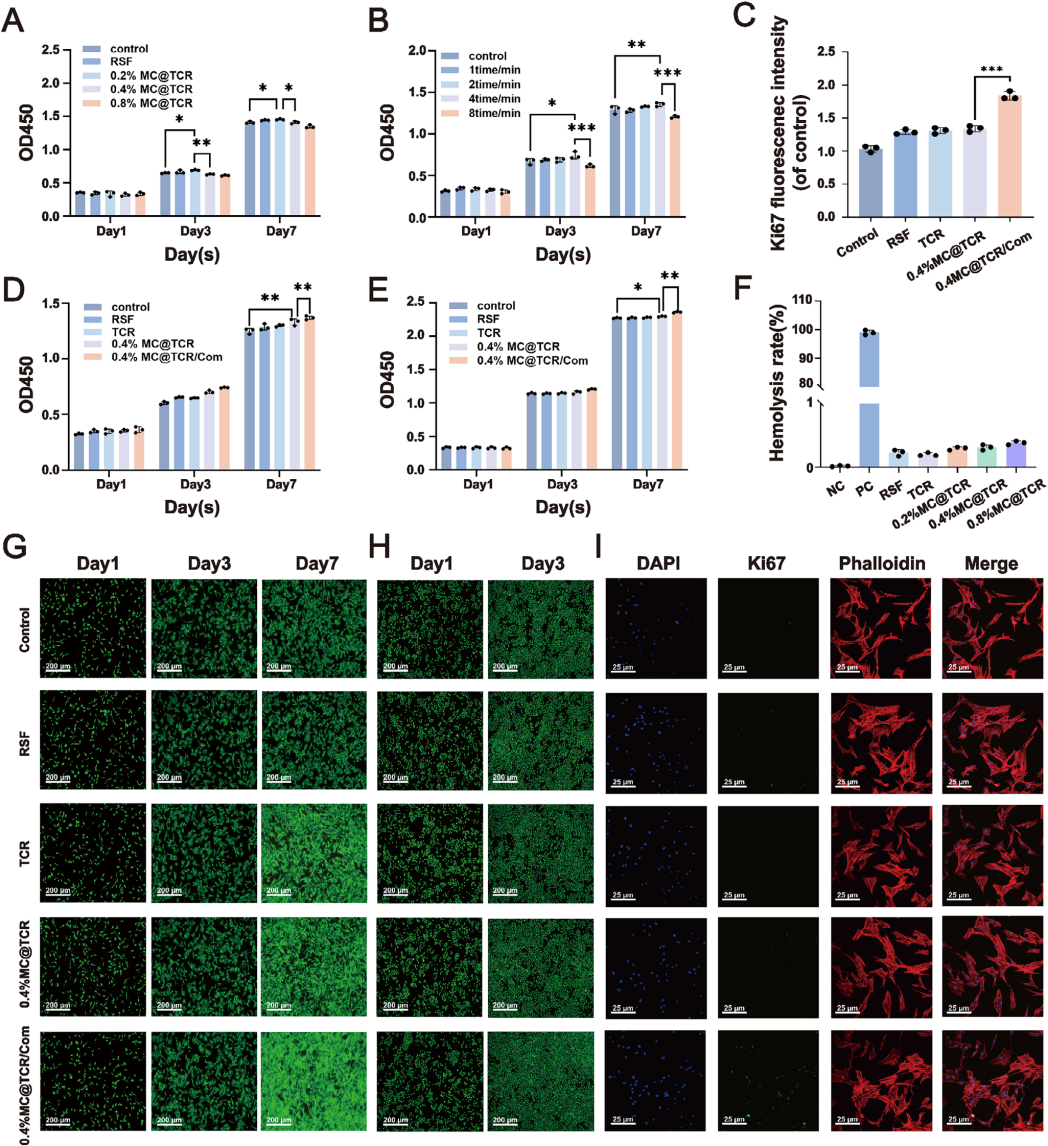
Biocompatibility evaluation of RSF/T‐CMC/MXene‐CNF hydrogels. (A) CCK‐8 assay of BMSCs cultured with extracts of control, RSF, and MC@TCR hydrogels containing different MXene‐CNF concentrations (0.2%, 0.4%, 0.8%). (B) CCK‐8 assay of BMSCs under different frequencies of piezoelectric stimulation (0, 1, 2, 4, and 8 times/min). (C) Ki67 immunostaining and semi‐quantitative fluorescence analysis of BMSCs in different groups. (D, E) CCK‐8 assays of BMSCs and HUVECs in control, RSF, TCR, 0.4% MC@TCR, and 0.4% MC@TCR/Com groups. (F) Hemolysis assay. (G, H) Live/Dead staining of BMSCs and HUVECs on different days. (I) Ki67 immunofluorescence staining of BMSCs in different hydrogel groups. ***p < 0.001, **p < 0.01, *p < 0.05, *n* = 3.

Since the piezoelectric hydrogel generates bioelectric cues after mechanical activation, the effect of hydrogel‐generated piezoelectric stimulation frequency on cell behavior was next evaluated. Importantly, in these in vitro experiments, cyclic compression was applied only to the hydrogel to activate its piezoelectric output, while cells were not directly subjected to mechanical compression or substrate deformation. Piezoelectric stimulation was applied at 0, 1, 2, 4, and 8 times/min (Figure 5B and Figures S12–S14), and the results showed that stimulation at 4 times/min produced the most pronounced increase in BMSC proliferation. Therefore, the 0.4% MXene‐CNF hydrogel under cyclic compression at 4 times/min was identified as the optimal condition for subsequent experiments.

To further assess the bioactive potential of this optimized hydrogel system, BMSCs and HUVECs were cultured in five groups: control, RSF, TCR, 0.4% MC@TCR, and 0.4% MC@TCR under compression (0.4% MC@TCR/Com). The 0.4% MC@TCR group served as the nonmechanically stimulated piezoelectric hydrogel control, in which cells were cocultured with the hydrogel without cyclic mechanical loading. The 0.4% MC@TCR/Com group represented the hydrogel mechanical activation‐induced piezoelectric stimulation group. CCK‐8 assays over multiple days (Figure 5D,E) demonstrated that the 0.4% MC@TCR/Com group exhibited the highest proliferation rates for both cell types, significantly outperforming the RSF and TCR groups. Live/Dead staining (Figure 5G,H and Figure S15) indicated that both BMSCs and HUVECs maintained high viability across all groups, and each group showed a gradual increase in cell density over time, confirming overall cytocompatibility of the hydrogels. Notably, cultures exposed to piezoelectric stimulation displayed a more pronounced rise in viable cell numbers compared with their unstimulated counterparts. Ki67 immunofluorescence staining (Figure 5C,I) and semi‐quantitative analysis further supported these findings, revealing that the 0.4% MC@TCR/Com group had the highest proliferative activity, highlighting the strong cytocompatibility and proliferative support offered by the hydrogel under piezoelectric stimulation.

Hemolysis represents another important indicator of biocompatibility [69, 70]. Biomaterials with a hemolysis ratio (HR) below 5% are generally considered acceptable for biomedical use [71]. Hemolysis testing (Figure 5F and Figure S16) showed that all hydrogel groups exhibited hemolysis ratios far below this threshold, demonstrating excellent blood compatibility. Collectively, these results show that the self‐reinforced piezoelectric hydrogel not only possesses intrinsic cytocompatibility but also actively enhances cell proliferation when subjected to dynamic mechanical‐electrical stimulation. This bioelectrical activation helps create a favorable microenvironment for osteogenic differentiation.

### 2.5 Osteogenic Differentiation Response of BMSCs Under Piezoelectric Stimulation

Alkaline phosphatase (ALP) activity and alizarin red S (ARS) staining were used to assess early‐ and late‐stage osteogenic differentiation of BMSCs, respectively [20, 72]. As shown in Figure 6A,B, after 7 and 14 days of culture, the 0.4% MC@TCR hydrogel under piezoelectric stimulation (0.4% MC@TCR/Com) displayed the strongest ALP staining and the most extensive mineralized nodule formation among all groups, clearly exceeding the RSF, TCR, and non‐stimulated 0.4% MC@TCR groups. These observations indicate that MXene‐CNF incorporation combined with cyclic mechanical activation synergistically enhances both osteogenic initiation and matrix mineralization. To further verify the osteoinductive effects at the molecular level, Western blot analysis was performed after 7 days of culture (Figure 6C,J–L and Figure S17). The expression of key osteogenic proteins—including osteopontin (OPN), runt‐related transcription factor 2 (RUNX2), and osteocalcin (OCN)—was markedly upregulated in the 0.4% MC@TCR/Com group compared with all other groups. Consistent with these protein trends, qRT‐PCR results (Figure 6F–I) showed that ALPL, OCN, OPN, and RUNX2 transcriptional levels were highest in the 0.4% MC@TCR/Com group, further confirming enhanced osteogenic gene activation. Immunofluorescence staining provided additional cellular‐level evidence. BMSCs grown on the 0.4% MC@TCR/Com hydrogels showed the strongest RUNX2 and COL1 fluorescence signals (Figure 6D,E,M,N), along with markedly higher semi‐quantitative fluorescence intensity compared with the other groups. These findings indicate that the dynamic piezoelectric cues produced by the self‐strengthening hydrogel environment effectively enhance osteogenic differentiation and matrix development. Collectively, these findings confirm that the 0.4% MC@TCR hydrogel under cyclic compression provides both structural and electrical cues that cooperatively induce strong osteogenic differentiation, suggesting that this piezoelectrically active hydrogel establishes a bioelectrically favorable microenvironment capable of accelerating bone regeneration.

**FIGURE 6:**
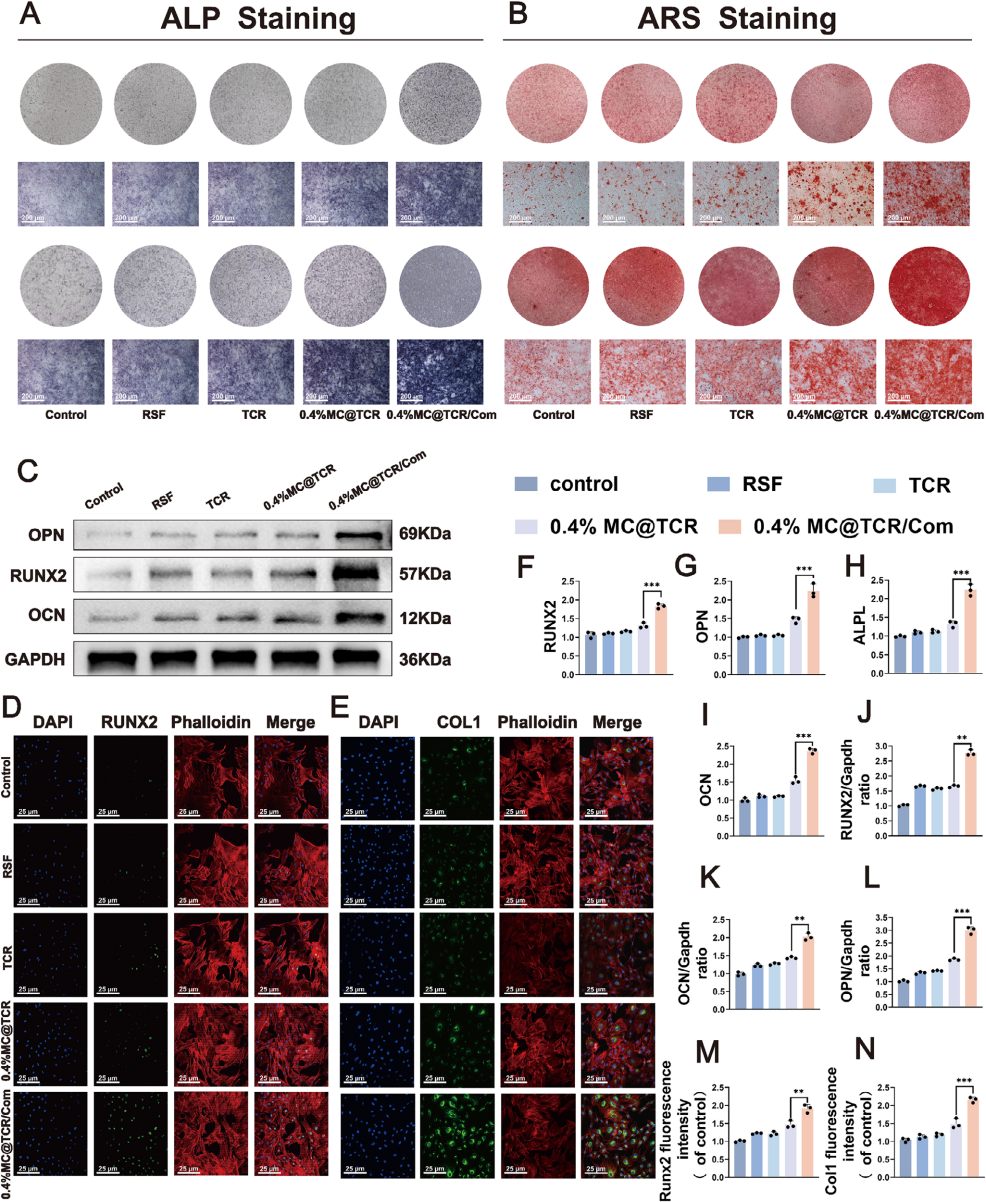
Osteogenic differentiation of BMSCs cultured with different hydrogels and piezoelectric stimulation. (A, B) ALP (day 7, 14) and ARS (day 14, 21) staining of BMSCs under five treatment conditions: control group, RSF group, TCR group, 0.4% MC@TCR group, and 0.4% MC@TCR/Com group. (C, J–L) Western blotting and quantitative analysis of osteogenic‐related proteins (OPN, Runx2, OCN) in BMSCs of each group after 7 days of culture. (F–I) Relative mRNA expression levels of osteogenic genes (RUNX2, OPN, ALPL, OCN) in BMSCs of each group after 7 days of culture. (D, M) Immunofluorescence staining and quantitative analysis of RUNX2 in BMSCs of each group after 7 days of culture. (E, N) Immunofluorescence staining and quantitative analysis of COL1 in BMSCs of each group after 7 days of culture. ***p < 0.001, **p < 0.01, *p < 0.05, *n* = 3.

### 2.6 Effect of MXene‐CNF/RSF/T‐CMC Piezoelectric Hydrogel With Compression Stimulation on BMSC Migration and HUVEC Angiogenesis In Vitro

Cell migration and angiogenesis are essential prerequisites for effective tissue regeneration, as these processes support cellular recruitment and vascular network formation at the defect site [73]. To examine whether piezoelectric stimulation enhances these regenerative behaviors, scratch wound assays were performed on HUVECs and BMSCs treated with different hydrogels. As shown in Figure 7A,B, the 0.4% MC@TCR hydrogel under compression (0.4% MC@TCR/Com) achieved the fastest wound closure among all groups, a result supported by quantitative analyses (Figure 7E,F). Increased BMSC migration suggests improved recruitment capacity toward the defect region, while enhanced HUVEC migration indicates elevated angiogenic activity—both critical for coordinated tissue repair [66, 74]. To further evaluate endothelial responsiveness under piezoelectric stimulation, a Transwell migration assay was performed on HUVECs (Figure 7D). In agreement with the scratch assay, the 0.4% MC@TCR/Com group showed the highest number of migrated cells, indicating that piezoelectric activation substantially enhances endothelial motility. Tube‐formation assays provided additional evidence of pro‐angiogenic potential: HUVECs exposed to 0.4% MC@TCR/Com formed more extensive capillary‐like networks—with increased junctions, longer tube length, and larger mesh structures—than all other groups (Figure 7C,J–L and Figure S18). At the molecular level, qRT‐PCR analysis showed significant upregulation of angiogenesis‐related genes (CD31, vWF, VEGF) after 3 days of stimulation (Figure 7G–I), confirming that piezoelectric activation promotes endothelial migration and vascular morphogenesis. Collectively, these results demonstrate that the self‐reinforcing piezoelectric hydrogel, when subjected to cyclic compression, enhances BMSC migration and markedly promotes endothelial angiogenesis in vitro, thereby establishing a pro‐regenerative microenvironment supportive of subsequent bone repair.

**FIGURE 7:**
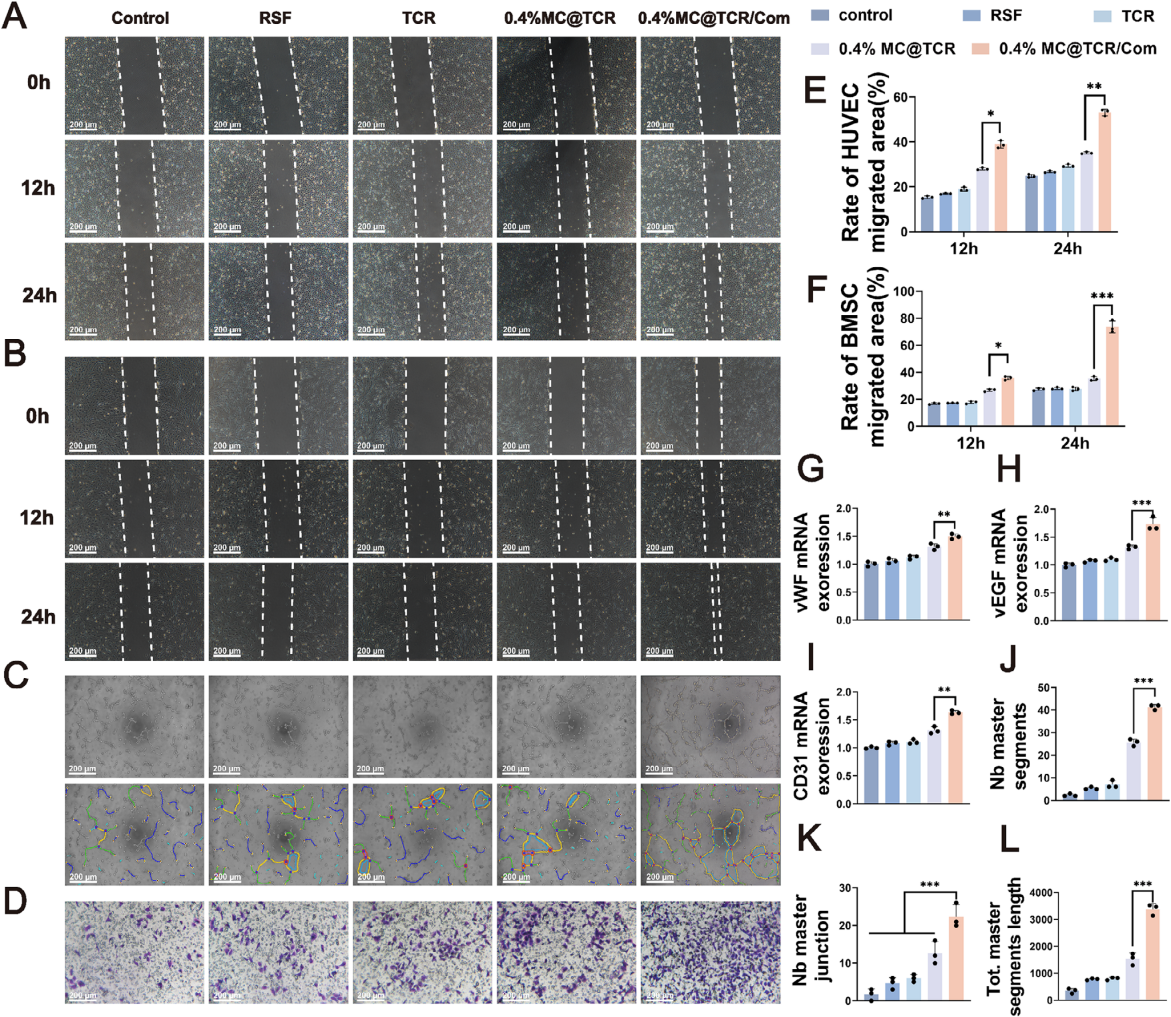
Migration, invasion, and angiogenesis assays of BMSCs and HUVECs under different treatments. (A, B) Scratch wound healing assay of HUVECs (A) and BMSCs (B) in the five groups: control, RSF, TCR, 0.4% MC@TCR, and 0.4% MC@TCR/Com.(C) Tube formation assay of HUVECs showing capillary‐like network formation under different treatments. (D) Transwell assay evaluating the invasion ability of HUVECs across the five groups. (E, F) Quantification of migration rates of HUVECs (E) and BMSCs (F). (G–I) Relative mRNA expression of angiogenesis‐related genes (vWF, VEGF, CD31) in HUVECs after 3 days of treatment. (J–L) Quantitative analysis of tube formation. ***p < 0.001, **p < 0.01, *p < 0.05, *n* = 3.

### 2.7 Piezoelectric Stimulation Activates Calcium Signaling Pathways to Drive Osteogenic Mechano‐transduction

To elucidate the molecular mechanism through which piezoelectric stimulation enhances osteogenesis, RNA sequencing was performed to compare transcriptomic profiles between piezoelectric and non‐piezoelectric groups. As illustrated in the heatmap (Figure 8A), piezoelectric stimulation produced a clear gene expression profile marked by increased expression of genes linked to calcium signaling and histone acetylation, reflecting coordinated activation of mechanosensitive and epigenetic regulatory mechanisms. GO enrichment analysis (Figure 8B) also showed that the calcium signaling pathway was one of the most prominently enriched pathways, together with other signaling routes related to osteogenesis. Consistently, the volcano plot (Figure 8C) identified 818 upregulated and 517 downregulated genes, with calcium channel‐related genes and osteogenic markers prominently enriched among the upregulated group. Gene Set Enrichment Analysis (GSEA) (Figure 8D,E and Figure S19) confirmed that both calcium signaling and osteogenic differentiation pathways were positively activated in response to piezoelectric cues.

**FIGURE 8:**
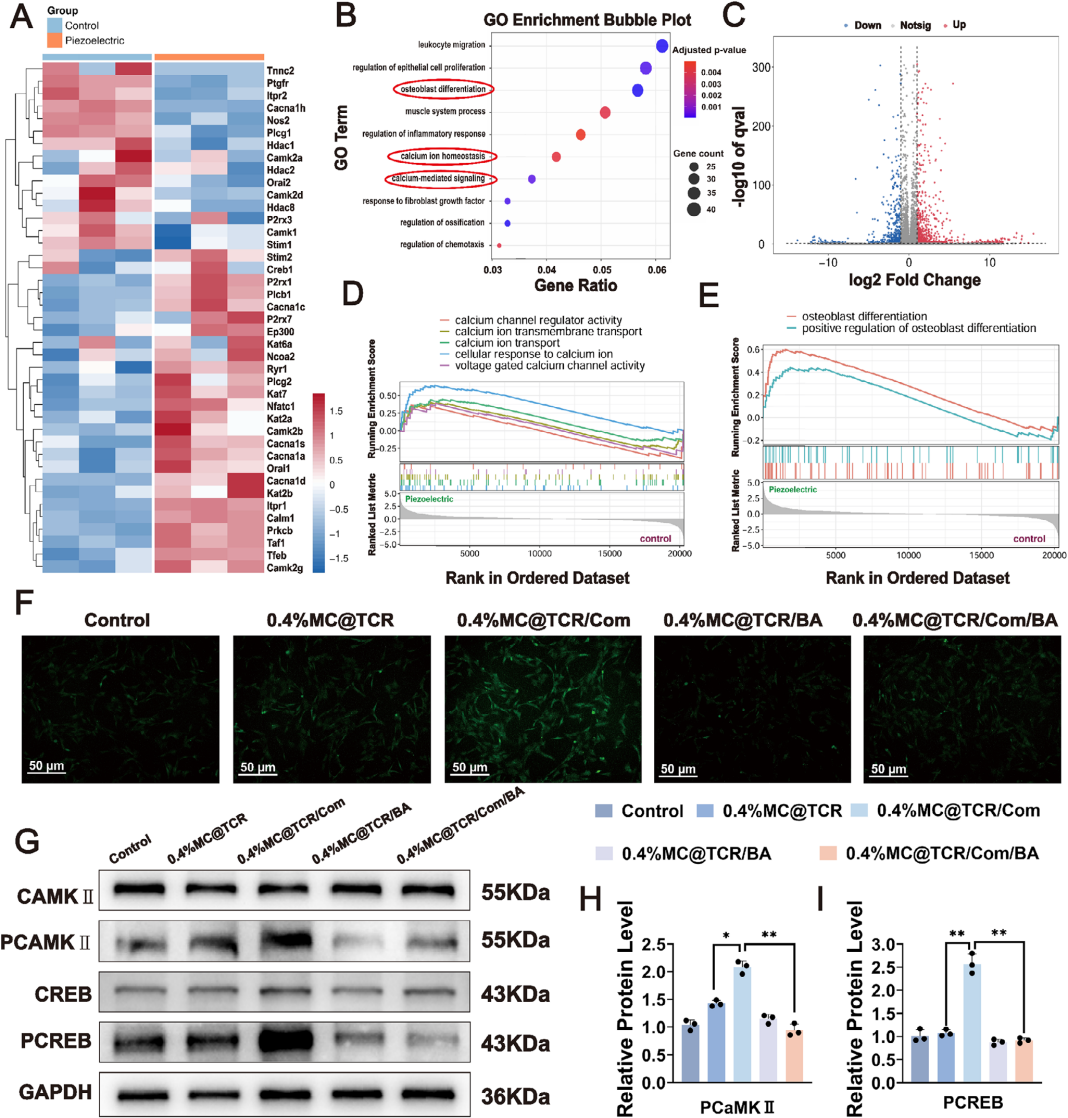
Transcriptomic analysis and calcium signaling validation under piezoelectric stimulation. (A) Heatmap showing differential gene expression between piezoelectric and non‐piezoelectric groups, highlighting activation of the calcium signaling pathway and upregulation of acetylation‐related genes. (B) GO signaling pathway enrichment analysis of RNA‐seq results. (C) Volcano plot showing upregulated and downregulated genes. (D, E) GSEA plots of calcium signaling and osteogenic differentiation pathways. (F) Fluorescence imaging of intracellular calcium influx in five groups: control, 0.4% MC@TCR, 0.4% MC@TCR/Com, 0.4% MC@TCR + BAPTA, and 0.4% MC@TCR/Com + BAPTA. (G–I) Western blot analysis of calcium signaling‐related proteins (p‐CaMKII, p‐CREB) across different groups. ***p < 0.001, **p < 0.01, *p < 0.05, *n* = 3.

To functionally validate these transcriptomic findings, intracellular Ca^2^^+^ levels were first assessed using Fluo‐4 AM fluorescence imaging after 30 min of hydrogel‐generated piezoelectric stimulation (Figure 8F). BMSCs in the 0.4% MC@TCR/Com group exhibited the highest Ca^2^^+^ fluorescence intensity, whereas Ca^2^^+^ chelation with BAPTA markedly suppressed this signal, indicating that hydrogel‐generated piezoelectric stimulation enhanced intracellular Ca^2^^+^ accumulation. To further clarify the temporal characteristics of Ca^2^^+^ signaling, live‐cell time‐series Fluo‐4 AM imaging was additionally performed during piezoelectric stimulation (Figure S20). The fluorescence intensity dynamically increased during stimulation, while BAPTA‐AM markedly attenuated this response, confirming the induction of Ca^2^^+^ transients by hydrogel‐generated piezoelectric cues. To further probe downstream events, Western blot analysis was conducted (Figure 8G–I and Figure S21). The 0.4% MC@TCR/Com group showed substantially elevated phosphorylation of CaMKII and CREB compared with non‐stimulated hydrogels, consistent with activation of the canonical calcium‐dependent signaling cascade. Together, these findings show that piezoelectric stimulation promotes osteogenic signaling by enhancing Ca^2+^ influx and triggering the CaMKII/CREB pathway, establishing a mechanistic link to the epigenetic and transcriptional activation observed in later experiments.

### 2.8 Piezoelectric Hydrogel Promotes Osteogenesis via Calcium Influx‐Mediated Histone Acetylation

To further determine whether the osteogenic improvement triggered by piezoelectric stimulation relies on a calcium‐dependent epigenetic process, early and late osteogenic markers were assessed under conditions with or without mechanical–electrical stimulation and with or without calcium chelation. ALP staining (day 7 and 14) and ARS staining (day 14 and 21) (Figure 9A,B) showed that piezoelectrically stimulated 0.4% MC@TCR hydrogels exhibited the strongest ALP activity and mineral deposition, whereas calcium chelation markedly suppressed these effects. These findings indicate that the osteogenic benefits of the hydrogel rely heavily on Ca^2+^ signaling. In line with this, immunofluorescence staining of H3K9ac (Figure 9C) revealed robust histone acetylation under piezoelectric stimulation, while calcium chelation substantially reduced the acetylation signal. Western blot analysis further supported this trend: piezoelectric stimulation significantly increased the expression of the epigenetic regulator P300 and elevated H3K9 acetylation, along with higher levels of osteogenic proteins OPN, RUNX2, and OCN (Figure 9D,E and Figure S22). In contrast, blocking Ca2+ influx abolished these increases, as confirmed by semi‐quantitative analysis (Figure 9F–J). Quantification of H3K9ac fluorescence intensity (Figure 9K) provided additional evidence that epigenetic activation is tightly dependent on calcium entry. To further determine whether H3K9 acetylation represents a dynamic epigenetic response to piezoelectric stimulation, H3K9ac levels were examined at 0, 1, 4, 8, 24, and 48 h after hydrogel‐generated piezoelectric stimulation (Figure S23). H3K9ac expression increased in a time‐dependent manner after stimulation, reached a relatively high level around 24 h, and then partially declined at 48 h. This temporal pattern suggests that piezoelectric stimulation induces a dynamic and partially reversible H3K9ac response rather than a sustained steady‐state modification or a single endpoint change. Together, these results demonstrate that the osteogenic effects of the 0.4% MC@TCR hydrogel arise from Ca^2+^‐dependent activation of P300‐mediated H3K9 acetylation, which in turn promotes transcriptional upregulation of osteogenic genes.

**FIGURE 9:**
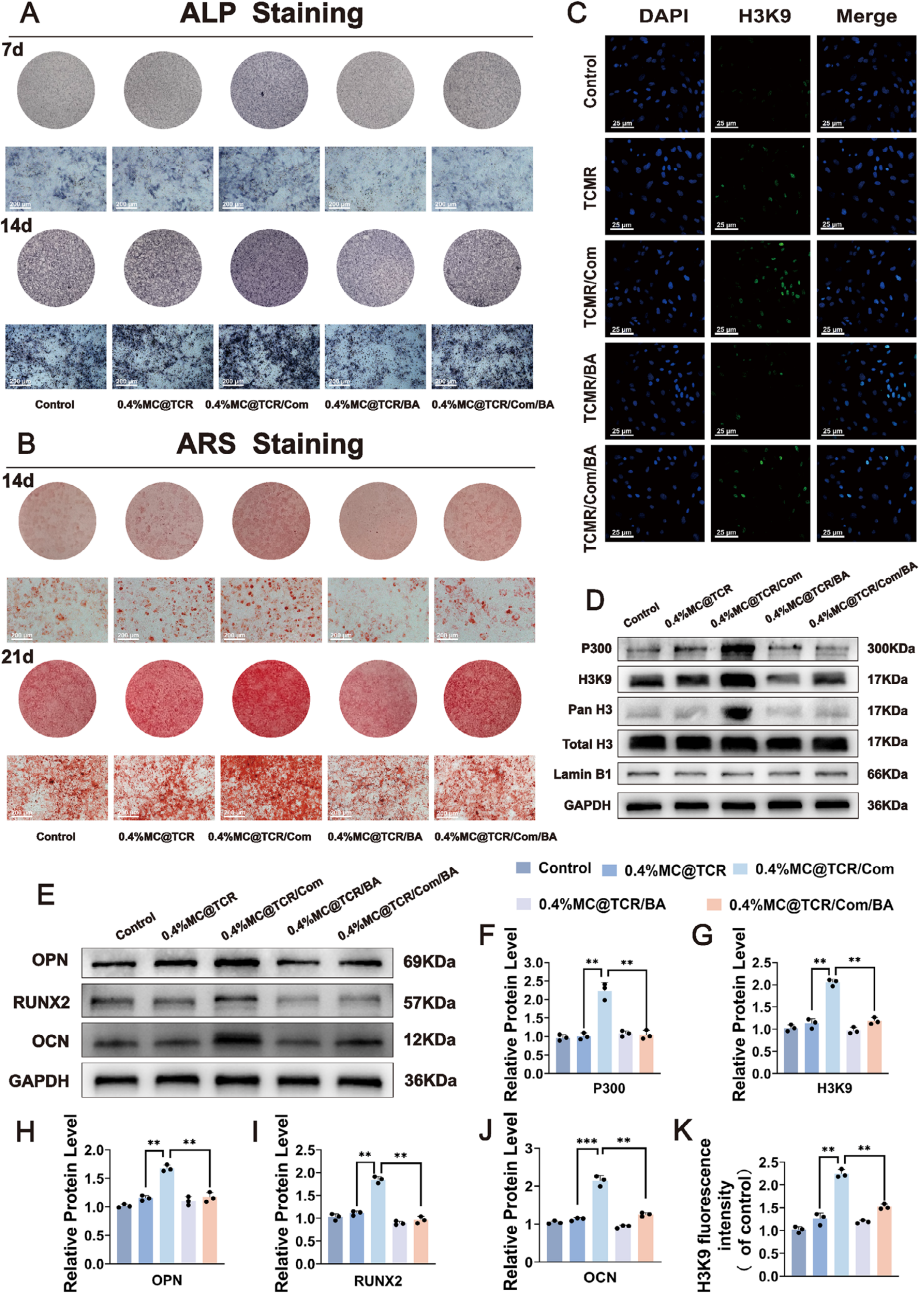
Epigenetic regulation and osteogenic differentiation under piezoelectric stimulation. (A, B) ALP (day 7, 14) and ARS (day 14, 21) staining of BMSCs under five treatment conditions: Control, 0.4% MC@TCR, 0.4% MC@TCR with compression (Com), 0.4% MC@TCR with BAPTA (BA), and 0.4% MC@TCR with combined compression and BAPTA (Com/BA). (C) H3K9 acetylation immunofluorescence staining showing epigenetic activation by piezoelectric stimulation and its suppression by calcium chelation. (D) Western blot bands of P300 and H3K9ac (with Pan‐H3, Total H3, Lamin B1, and GAPDH as controls). (E) Western blot bands of osteogenic proteins (OPN, RUNX2, OCN; normalized to GAPDH). (F, G) Quantitative analysis of P300 and H3K9ac expression levels. (H–J) Quantitative analysis of OPN, RUNX2, and OCN. (K) Quantification of H3K9ac immunofluorescence intensity. ***p < 0.001, **p < 0.01, *p < 0.05, *n* = 3.

### 2.9 P300‐Mediated Histone Acetylation Serves as a Downstream Effector of Piezoelectric‐Induced Calcium Signaling

To determine whether the osteogenic effects observed under piezoelectric stimulation result from calcium‐dependent epigenetic regulation, osteogenic differentiation and histone acetylation were examined with and without P300 inhibition. As shown by ALP staining at days 7 and 14 and ARS staining at later stages (Figure 10A,B), piezoelectric stimulation markedly enhanced early alkaline phosphatase activity and subsequent mineral deposition compared with non‐stimulated hydrogels. However, pharmacological inhibition of P300 substantially decreased both effects—even when piezoelectric cues were present—indicating that P300 activity is essential for the full osteogenic response. In agreement with these functional observations, immunofluorescence analysis of H3K9ac (Figure 10C) showed a marked rise in histone acetylation under piezoelectric stimulation, whereas inhibition of P300 substantially diminished this signal. Western blot results (Figure 10D,E and Figure S24) further indicated that piezoelectric stimulation increased P300 and H3K9ac levels along with the osteogenic markers RUNX2, OPN, and OCN, while suppression of P300 eliminated both histone acetylation and subsequent osteogenic protein expression. Semi‐quantitative evaluations (Figure 10F–J) and fluorescence measurements of H3K9ac (Figure 10K) consistently supported these results. Together, these results indicate that P300‐mediated H3K9 acetylation acts as a key downstream effector of Ca2+ influx during piezoelectric stimulation, functioning as an epigenetic switch that converts mechanical‐electrical signals into transcriptional activation of osteogenic programs. This mechanistic link between calcium signaling, chromatin remodeling, and osteogenic gene expression motivated further investigation into whether piezoelectric stimulation enriches H3K9ac at specific osteogenic gene loci.

**FIGURE 10:**
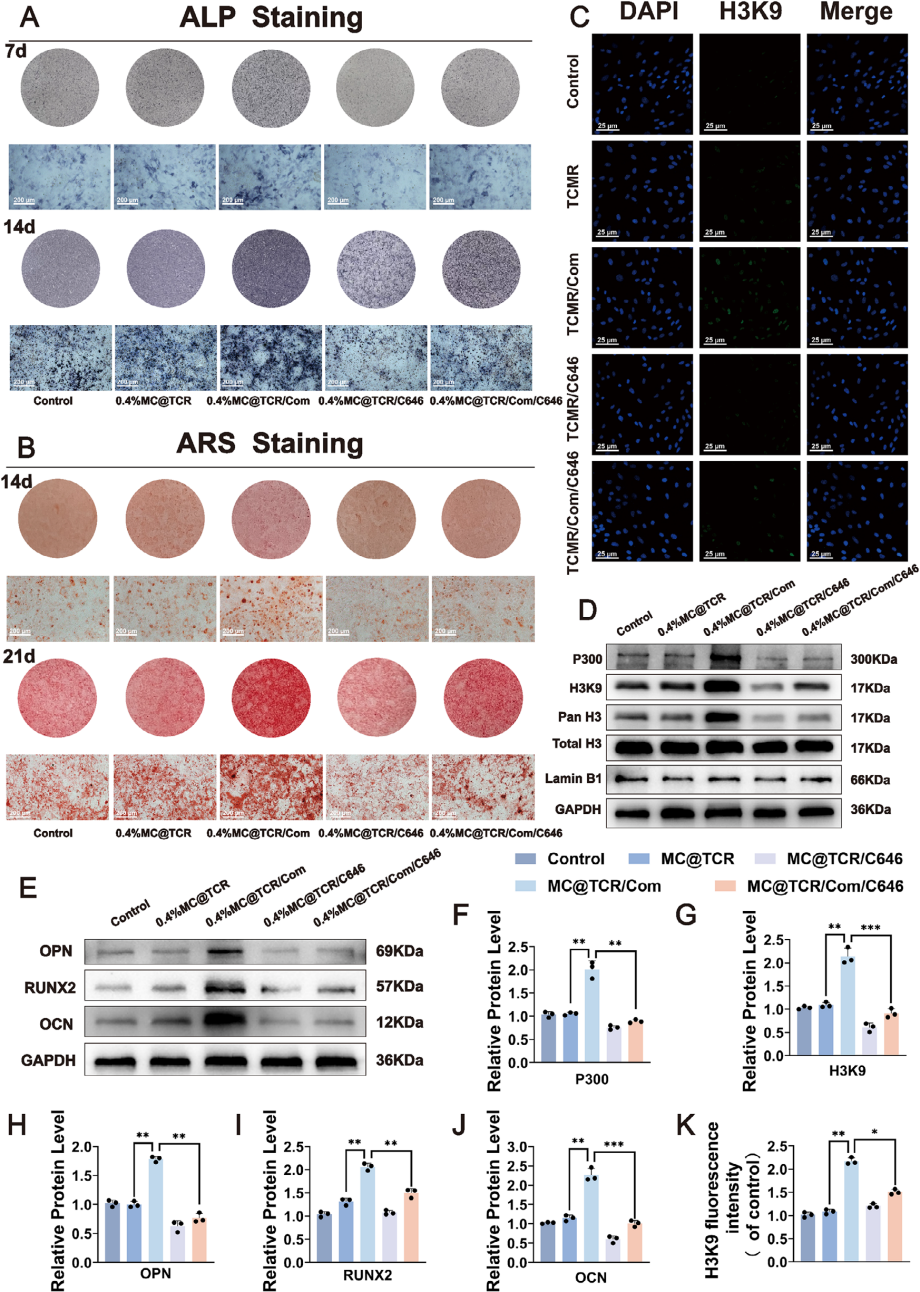
Epigenetic regulation of osteogenesis under piezoelectric stimulation with acetylation inhibition. (A, B) ALP staining (day 7, 14) and ARS staining (day 14, 21) of BMSCs cultured under five treatment conditions: Control, 0.4% MC@TCR, 0.4% MC@TCR with compression (Com), 0.4% MC@TCR with the P300 inhibitor C646 (C646), and 0.4% MC@TCR with combined compression and C646 inhibition (Com/C646), showing both early and late osteogenic responses. (C) Immunofluorescence staining of H3K9 acetylation showing epigenetic activation by piezoelectric stimulation and its suppression by P300 inhibition. (D) Western blot bands of P300 and H3K9ac, with Pan H3, Total H3, and Lamin B1 as nuclear loading controls and GAPDH as the cytoplasmic control. (E) Western blot bands of RUNX2, OPN, and OCN normalized to GAPDH. (F–G) Quantitative analysis of P300 and H3K9ac expression levels. (H–J) Quantitative analysis of RUNX2, OPN, and OCN. (K) Quantification of H3K9ac fluorescence intensity showing that P300 inhibition attenuates piezoelectric‐induced histone acetylation. ***p < 0.001, **p < 0.01, *p < 0.05, *n* = 3.

### 2.10 Epigenomic Profiling Identifies PTH1R as a Downstream Effector of H3K9ac Under Piezoelectric Stimulation

Having established that piezoelectric stimulation elevates intracellular Ca^2+^ and activates the P300–H3K9ac signaling axis, we next examined whether these chromatin changes extend to genome‐wide transcriptional activation. CUT&Tag profiling of H3K9ac revealed a clear global increase in acetylation under piezoelectric stimulation (Figure 11A), reflected by the warmer color patterns across gene loci. To pinpoint specific chromatin regions affected by stimulation, a volcano plot of differential H3K9ac peaks was generated (Figure 11B). Distinct clusters of significantly upregulated (red) and downregulated (blue) peaks were observed, indicating that piezoelectric stimulation selectively modifies defined genomic regions rather than inducing a uniform chromatin shift. This selectivity suggests targeted epigenetic remodeling rather than random changes in acetylation. To interpret the functional significance of these regions, genes upregulated exclusively in the CUT&Tag dataset were analyzed. GO enrichment (Figure 11C) showed strong enrichment in ossification, extracellular matrix organization, and calcium‐responsive pathways, suggesting that piezoelectric stimulation initiates osteogenic epigenetic priming before full transcriptional responses develop. To identify genes that were both epigenetically activated and transcriptionally upregulated, CUT&Tag results were integrated with RNA‐seq, and overlapping upregulated genes were extracted (Figure 11D). Enrichment analysis (Figure 11G) highlighted processes central to bone regeneration—including bone remodeling, ossification, ECM regulation, and calcium‐mediated signaling—demonstrating convergence between chromatin activation and transcriptomic remodeling. Within this intersecting gene set, PTH1R emerged as the most compelling target, appearing at the intersection of osteogenic and calcium‐responsive pathways and showing simultaneous elevation at both the chromatin (H3K9ac) and transcriptional levels. Track visualization (Figure 11E) confirmed substantial H3K9ac enrichment at the PTH1R promoter together with increased transcription, while motif analysis (Figure 11F) identified enriched transcription‐factor binding motifs, supporting active promoter engagement. To verify that PTH1R induction depends on P300‐mediated acetylation, we assessed PTH1R expression under piezoelectric stimulation with or without P300 inhibition. Piezoelectric stimulation markedly elevated PTH1R mRNA and protein levels, whereas P300 inhibition substantially reduced this response (Figure 11H–J and Figure S25), placing PTH1R downstream of the Ca^2+^–P300–H3K9ac pathway. Finally, functional assays confirmed that PTH1R is required for the osteogenic phenotype: pharmacological blockade of PTH1R decreased RUNX2, OPN, and OCN expression and significantly reduced ALP activity and matrix mineralization, even under piezoelectric stimulation (Figure 11K–Q and Figure S25). Together, these results establish a coherent epigenetic‐transcriptional cascade in which piezoelectric stimulation drives Ca^2+^ influx, activates P300‐dependent H3K9 acetylation, promotes PTH1R transcription, and ultimately enhances osteogenic gene expression, linking mechanical‐electrical cues to chromatin remodeling and functional bone regeneration.

**FIGURE 11:**
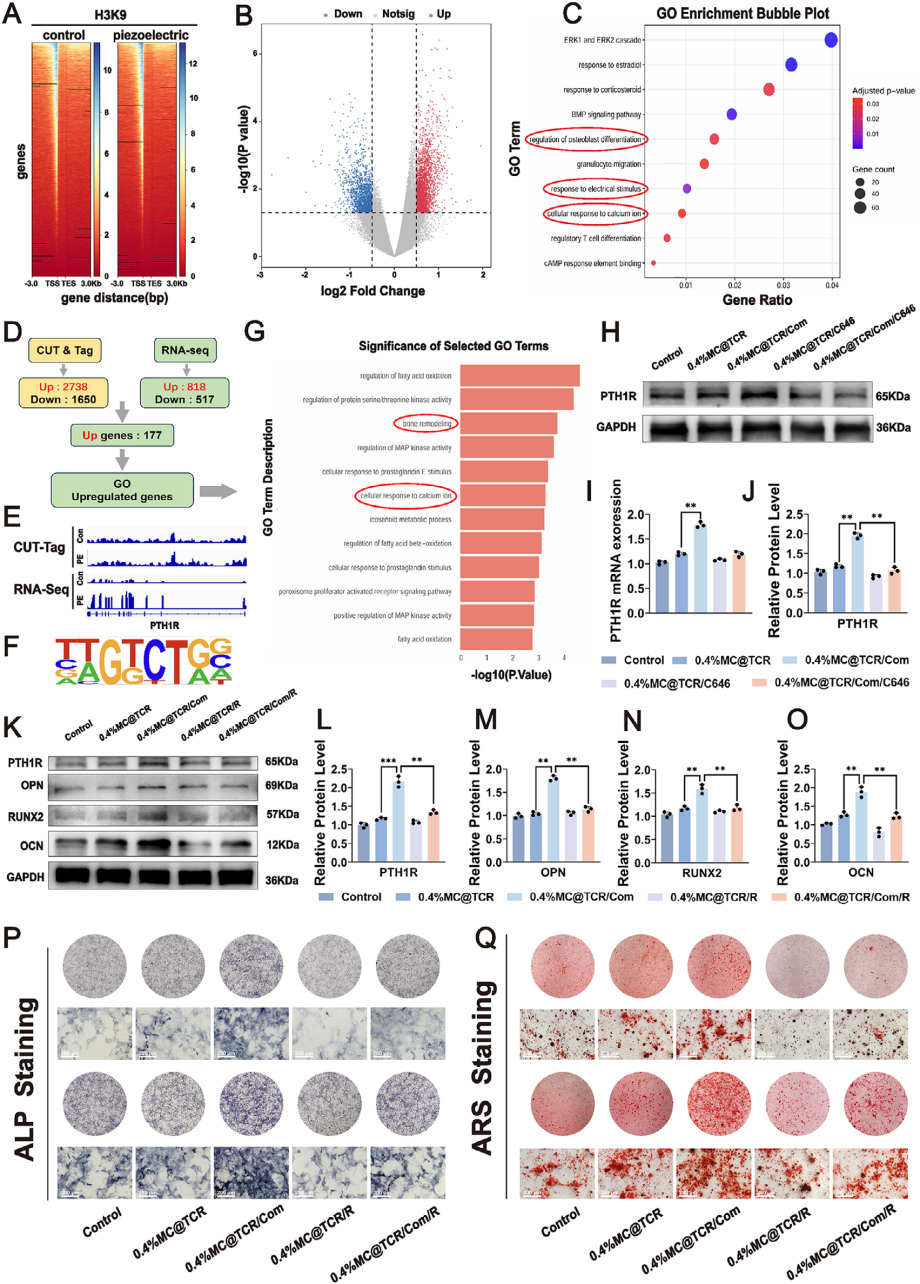
Identification of PTH1R as an epigenetic downstream target of piezoelectric stimulation. (A) CUT&Tag heatmap showing enhanced H3K9ac enrichment under piezoelectric stimulation. (B) Volcano plot of differential CUT&Tag peaks. (C) GO analysis of genes upregulated by CUT&Tag. (D, G) GO analysis of overlapping genes identified in both CUT&Tag and RNA‐seq datasets. (E) Genome browser tracks showing increased CUT&Tag and RNA‐seq signals at the PTH1R locus. (F) Sequence logo of enriched DNA motifs in H3K9ac peaks at the PTH1R region. (H) Western blot analysis of PTH1R expression in five groups: Control, 0.4% MC@TCR, 0.4% MC@TCR with compression (Com), 0.4% MC@TCR with C646, and 0.4% MC@TCR with combined compression and C646 inhibition (Com/C646). (I, J) Relative mRNA and semi‐quantitative protein expression of PTH1R. (K) Western blot analysis of PTH1R, OPN, RUNX2, and OCN in five groups: Control, 0.4% MC@TCR, 0.4% MC@TCR with compression (Com), 0.4% MC@TCR with PTH1R inhibitor (R), and 0.4% MC@TCR with combined compression and inhibitor (Com/R). (L–O) Semi‐quantitative analysis of PTH1R, OPN, RUNX2, and OCN. (P, Q) ALP and ARS staining showing that PTH1R inhibition reduces piezoelectric‐induced osteogenic differentiation. ***p < 0.001, **p < 0.01, *p < 0.05, *n* = 3.

### 2.11 Piezoelectric Hydrogel Promotes Bone Regeneration in a Rat Femoral Defect Model

To further substantiate the in vitro results and verify the bone‐repair capability of the piezoelectric hydrogel in vivo, a rat femoral defect model was created. As depicted in Figure 12A, the procedure involved skin preparation and disinfection, formation of a standardized critical‐sized defect, in situ hydrogel injection, and final wound closure. Micro‐CT scans were conducted at 6 and 12 weeks after surgery to evaluate new bone regeneration across four groups: Control, RSF, TCR, and 0.4% MC@TCR. As presented in Figure 12B, the 0.4% MC@TCR group exhibited substantial bone ingrowth and progressive defect closure, while the control and RSF groups showed only limited repair. To provide a more direct visualization of the osteogenic process, representative Micro‐CT three‐dimensional reconstruction images of the defect regions were added in Figure S26, showing progressive new bone ingrowth, defect filling, and trabecular‐like bone formation within the femoral defect area. Quantitative Micro‐CT analysis (Figure 12C–F) further demonstrated that bone mineral density (BMD), bone volume fraction (BV/TV), trabecular thickness (Tb.Th), and trabecular number (Tb.N) were significantly higher in the 0.4% MC@TCR group at both 6 and 12 weeks, confirming superior osteoconductive and osteoinductive capacity. To assess dynamic mineralization, Alizarin Red S and Calcein double labeling was performed (Figure 12G–I). Fluorochrome‐labeled deposition showed a markedly higher bone formation rate in the piezoelectric hydrogel group, supporting continuous bone remodeling during healing. These in vivo results collectively indicate that the self‐reinforcing piezoelectric hydrogel establishes a favorable osteogenic microenvironment, translating its mechanobiological activation into robust bone regeneration, which motivated further investigation into the cellular and molecular events underlying this regenerative process in subsequent histological and immunohistochemical analyses.

**FIGURE 12:**
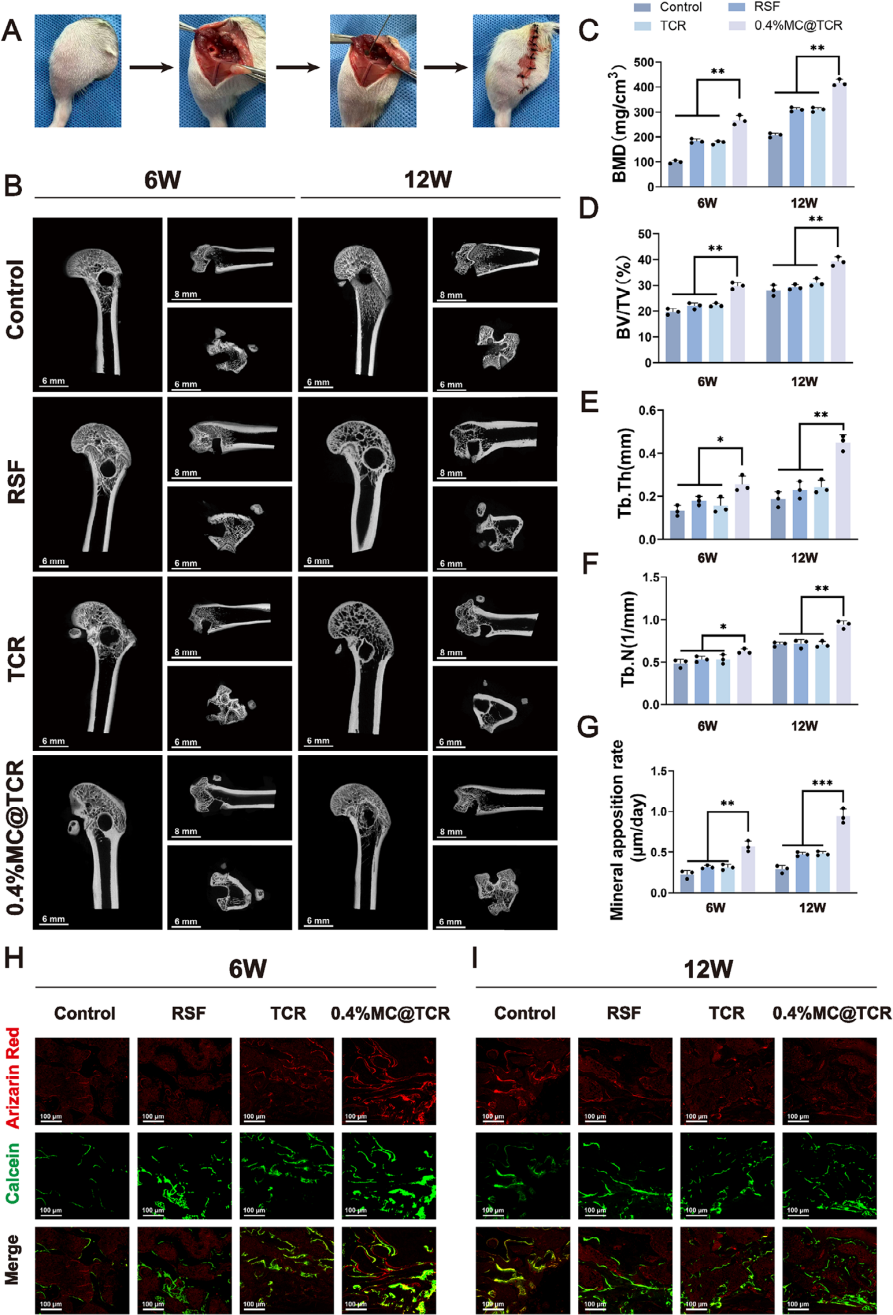
In vivo bone regeneration evaluation in a rat femoral defect model. (A) Schematic illustration of the surgical procedure, including skin preparation and disinfection, drilling of a standardized femoral defect, in situ injection of the hydrogel, and wound closure. (B) Representative Micro‐CT images of defect regions at 6 and 12 weeks post‐surgery in four groups—Control (empty defect), RSF hydrogel, TCR hydrogel, and 0.4% MC@TCR piezoelectric hydrogel—showing time‐dependent new bone formation. (C–F) Quantitative Micro‐CT analysis of bone regeneration, including bone mineral density (BMD), bone volume fraction (BV/TV), trabecular thickness (Tb.Th), and trabecular number (Tb.N). (G) Quantitative evaluation of MAR at 6 and 12 weeks after hydrogel implantation compared with the defect‐only control group. (H, I) Representative Alizarin Red S and Calcein double‐labeling images at 6 and 12 weeks, showing mineral deposition and dynamic bone formation across the four groups. ***p < 0.001, **p < 0.01, *p < 0.05, *n* = 3.

### 2.12 Histological Assessment Reveals Accelerated Bone Regeneration and Matrix Maturation Induced by Piezoelectric Hydrogel

To further assess the quality and maturity of newly formed bone in vivo, histological staining was performed on femoral defect sections harvested at 6 and 12 weeks post‐implantation. H&E, TB, VG, and Masson's trichrome staining (Figure 13A,B) showed a time‐dependent progression of bone regeneration in all groups. Minimal bone formation and extensive fibrous tissue were observed in the control group, while the RSF and TCR groups presented partial bone ingrowth with limited matrix organization. In contrast, the 0.4% MC@TCR group displayed extensive new bone deposition, well‐developed trabecular architecture, and dense collagen fiber alignment, indicating superior osteoconductivity and defect‐bridging capability. These observations are consistent with the Micro‐CT analyses and further confirm that piezoelectric stimulation enhances intramembranous ossification and matrix maturation. To determine whether epigenetic and osteogenic signaling pathways were activated in vivo, immunohistochemical staining for H3K9ac, PTH1R, Collagen I, and OCN was performed at 12 weeks (Figure 13C–F). The 0.4% MC@TCR group showed markedly elevated H3K9 acetylation and PTH1R expression, reflecting sustained epigenetic activation and calcium‐responsive signaling under piezoelectric stimulation. Correspondingly, increased Collagen I and OCN staining confirmed active osteogenic transcription and matrix mineralization. Systemic biosafety was also evaluated. Serum biochemical indicators measured at 12 weeks post‐implantation (Figure S27) showed no abnormalities in hepatic or renal function across all groups, confirming metabolic safety. H&E staining of major organs—including heart, liver, spleen, lung, and kidney—revealed no inflammatory infiltration or tissue damage (Figure S28), demonstrating excellent in vivo biocompatibility and biosafety of the hydrogels. This favorable biosafety profile may be partly related to the stable incorporation of MXene‐CNF nanosheets within the RSF/T‐CMC hydrogel network. Therefore, the low‐content MXene‐CNF nanosheets in the present RSF/T‐CMC network may be protected from burst release and direct tissue exposure, contributing to the favorable in vivo biosafety. To further verify whether the hydrogel could generate electrical signals under bone‐like mechanical loading, an ex vivo porcine bone defect model was established. The 0.4% MC@TCR hydrogel was implanted into a defect created in porcine bone, and electrodes were placed on both sides of the hydrogel to collect the electrical output during cyclic compression. A detectable and repeatable voltage signal was recorded from the hydrogel‐implanted bone construct, confirming that the piezoelectric hydrogel retained electromechanical activity after implantation into a bone defect‐like microenvironment. These results provide direct ex vivo evidence that the implanted hydrogel can convert mechanical loading into electrical output, supporting the feasibility of piezoelectric activation in vivo under physiological mechanical stimulation (Figure S29). Together, these findings demonstrate that the piezoelectric hydrogel not only accelerates bone defect repair but also activates epigenetic and molecular pathways driving osteogenesis in vivo, while maintaining high systemic safety and tissue compatibility.

**FIGURE 13:**
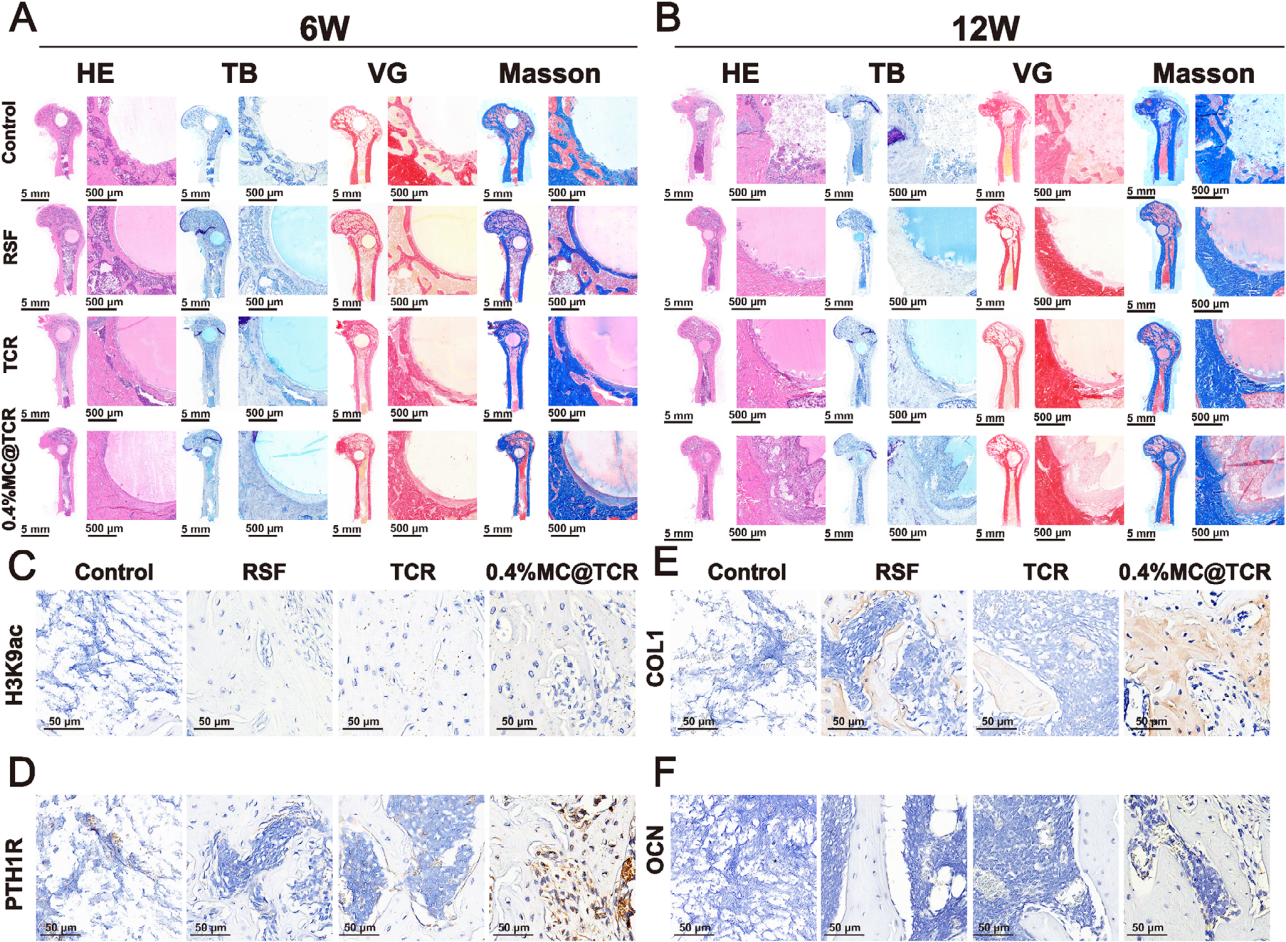
Histological and immunohistochemical evaluation of in vivo bone regeneration. (A, B) H&E, TB, VG, and Masson's trichrome staining of femoral defect sections at 6 and 12 weeks in the Control, RSF, TCR, and 0.4% MC@TCR groups, showing progressive new bone formation, collagen deposition, and defect bridging. (C–F) Immunohistochemical staining at 12 weeks for H3K9ac, PTH1R, Collagen I, and OCN in the four groups.

## 3 Conclusion

In this study, we developed an injectable and self‐reinforced piezoelectric hydrogel that integrates mechanical adaptability, electrical responsiveness, and epigenetic regulatory capacity for bone regeneration in irregular femoral defects. The hydrogel, composed of RSF, MXene‐CNF, and T‐CMC, undergoes progressive molecular rearrangement that enhances β‐sheet crystallinity and piezoelectric output over time. This dynamic self‐strengthening behavior provides both mechanical stability and sustained bioelectrical stimulation. The piezoelectric cues triggered Ca2+ entry and activation of the CaMKII/CREB/P300 axis, elevated H3K9 acetylation, and enhanced the expression of essential osteogenic genes, with PTH1R functioning as a key mediator linking electrical signals to epigenetic regulation. In the rat femoral defect model, the optimized 0.4% MC@TCR hydrogel substantially accelerated bone regeneration, improving bone mineral density, trabecular formation, collagen accumulation, and the levels of H3K9ac and PTH1R. Overall, these results demonstrate an effective approach for bone repair: a self‐reinforcing piezoelectric scaffold that not only fills the defect but also remodels the osteogenic microenvironment through calcium‐dependent epigenetic control, providing strong translational promise for complex long‐bone reconstruction.

## 4 Experimental Section

### 4.1 Materials

Anhydrous sodium carbonate, horseradish peroxidase, carboxymethyl cellulose, tyramine, H_2_O_2_, and CNF were purchased from Aladdin. Lithium bromide was purchased from Roan's Reagent. α‐MEM medium, fetal bovine serum, and penicillin‐streptomycin were purchased from Gibco (Massachusetts, USA). Rats were purchased from SPF (Beijing, CHINA). Dialysis membrane (molecular weight cutoff = 14 000) was purchased from BBI (Shanghai, China). Antibodies against GAPDH, RUNX2, Lamin B1, and PTH1R were purchased from Proteintech (Chicago, USA). Antibodies against OPN and OCN were purchased from Abclonal (Minnesota, USA). Antibodies against Collagen 1, P300, H3K9, and total H3 were purchased from Abcam (Massachusetts, USA). Antibodies against CAMK, PCAMK, and CREB, PCREB were purchased from Selleck (Shanghai, CHINA). CCK‐8 kit, ALP staining kit, Alizarin Red S staining kit (ARS), Live/Dead viability assay kit, Ki67 immunofluorescence staining kit, Fluo‐4 AM kit were purchased from Beyotime (Beijing, China). BAPTA‐AM, C646 were purchased from Sigma–Aldrich (Massachusetts, USA). PTH1R antagonist was purchased from MCE (New Jersey, USA)

### 4.2 Preparation of RSF Solutions

Silk fibers were degummed in 0.5% sodium carbonate at 100°C (2 × 30 min), rinsed, dried at 60°C, and dissolved in 9.3 M LiBr at 60°C for 1 h. After filtration, the solution was dialyzed (MWCO 14 kDa) against deionized water for 3 days and concentrated with PEG to obtain regenerated silk fibroin (RSF).

### 4.3 Preparation of MXene‐CNF

Ti_3_AlC_2_ powder was first etched in a mixed solution of 12 M HCl and LiF at 35°C for 24 h, selectively removing the Al layer to obtain multilayer Ti_3_C_2_T_x_. The etched product was repeatedly washed to neutrality and then delaminated through Li^+^ intercalation assisted by mild sonication, yielding single‐ or few‐layer MXene nanosheets. The resulting MXene nanosheets were subsequently mixed with cellulose nanofibers (CNF). The abundant hydroxyl groups (─OH) on CNF formed dense hydrogen‐bonding networks with the surface terminations (─O/─OH) of MXene, producing a stable and well‐dispersed MXene‐CNF composite.

### 4.4 Preparation of T‐CMC

T‐CMC was synthesized via an EDC/NHS‐mediated coupling reaction. Briefly, CMC (0.2% w/v) was dissolved in deionized water, followed by the sequential addition of EDC, NHS, and tyramine. The reaction was stirred at room temperature for 24 h. The crude product was dialyzed against deionized water (MWCO 14 kDa) for 48 h with frequent water changes, and then concentrated using PEG (MW 20 000) for 24 h. The final T‐CMC solution was stored at 4°C until use. Successful grafting of tyramine was confirmed by ^^1^H NMR spectroscopy.

### 4.5 Fabrication of Composite Hydrogels

RSF solution (10% w/v) was mixed with MXene‐CNF suspension, followed by addition of T‐CMC (0.3% w/v) and homogenization. Gelation was initiated with HRP (900 U·mL^−^^1^) and H_2_O_2_ (0.6% v/v) under mild stirring. The formed hydrogels were subsequently incubated at 37°C to allow progressive self‐reinforcement. Different groups were prepared by varying MXene‐CNF content.

### 4.6 Physicochemical and Electromechanical Characterization of RSF/T‐CMC/MXene‐CNF Piezoelectric Hydrogel

The structural properties of RSF/T‐CMC/MXene‐CNF hydrogels were characterized by Fourier transform infrared spectroscopy (FTIR, Nicolet iS50, USA) to identify characteristic functional groups and by x‐ray diffraction (XRD, D/Max2550, Japan) to evaluate β‐sheet crystallinity and MXene interlayer structure. Surface elemental composition and chemical states were analyzed by x‐ray photoelectron spectroscopy (XPS, ESCALAB 250Xi, USA). The morphology of CNF, MXene, MXene‐CNF, and composite hydrogels was examined using field‐emission scanning electron microscopy (FE‐SEM, Gemini SEM 500, Germany) and transmission electron microscopy (TEM, JEM‐2100, Japan), while MXene‐CNF distribution within the hydrogel matrix was confirmed by EDS mapping. Rheological behavior, including gelation kinetics and sol–gel transition, was measured using a rheometer (MCR302, Anton Paar, Austria). Compressive strength and modulus were determined using a universal testing machine (CMT2503, USA), and Ashby plots were constructed for comparison with reported injectable piezoelectric hydrogels. Dielectric constant and dielectric loss were measured using a precision LCR meter (E4980A, Keysight, USA). For electromechanical evaluation, hydrogels were molded into cylindrical disks, sandwiched between copper foil electrodes, and subjected to cyclic compression; real‐time voltage and current were recorded using a Keithley DMM6500. Pressure‐dependent performance (0.06–0.15 MPa) was assessed to evaluate sensitivity and stability. Nanoscale piezoelectric behavior, including amplitude and phase responses, was characterized via piezoresponse force microscopy (PFM, Bruker Dimension Icon, Germany) under a 10 V AC excitation. For practical sensing demonstrations, hydrogel‐based piezoelectric devices were attached to finger joints to monitor motion‐induced electrical signals. In vitro swelling and degradation assays were performed by immersing hydrogels in PBS at 37°C. At predetermined time points, samples were removed, gently blotted or lyophilized as required, and weighed to calculate the swelling ratio or remaining weight. To evaluate in vivo mechanical and piezoelectric stability, implanted 0.4% MC@TCR hydrogels were harvested at 0, 14, and 28 days. Retrieved samples were rinsed with PBS and subjected to compressive testing and voltage output measurement under cyclic compression. Tensile tests were conducted using a universal testing machine. Hydrogel samples were stretched until failure, and tensile strength and elongation at break were determined from the stress–strain curves. Stress relaxation tests were performed by compressing the hydrogels to 20% strain and maintaining this strain for 600 s. The stress was recorded over time and normalized to the initial stress.

### 4.7 Cell Viability and Proliferation

Ring‐shaped RSF/T‐CMC/MXene‐CNF hydrogels were mechanically activated using a custom‐built piezoelectric actuator (10 N, 4 cycles/min, 6 h/day) to generate electrical stimulation. BMSCs and HUVECs were cultured in the central region of the ring‐shaped hydrogels, rather than on the mechanically compressed hydrogel surface, and were exposed to the electrical microenvironment generated by the activated piezoelectric hydrogels. Thus, cyclic compression was applied only to the hydrogel samples, while the cells were not directly compressed, seeded on a deformable compressed substrate, or exposed to hydrogel‐mediated mechanical deformation. For cytocompatibility, hydrogel extracts were prepared by incubating samples in α‐MEM at 37°C for 24 h. Cell viability of BMSCs and HUVECs was assessed by CCK‐8 (Beyotime, China) at days 1, 3, 5, and 7, while Live/Dead staining (Calcein‐AM/PI, Beyotime, China) was performed and imaged by fluorescence microscopy (Olympus IX71, Japan).

### 4.8 Osteogenic Induction and Related Protein Analysis

BMSCs (1 × 10^5^ cells/well) were seeded in six‐well plates and cultured in complete growth medium until reaching 70%–80% confluency. The medium was then replaced with osteogenic induction medium consisting of α‐MEM supplemented with 0.05 mM vitamin C, 10 mM β‐glycerophosphate, and 10 nM dexamethasone. Subsequently, the cells were cocultured with the hydrogels under hydrogel‐generated piezoelectric stimulation according to the aforementioned culture protocol. The osteogenic medium was refreshed every 3 days. Osteogenic differentiation was assessed by BCIP/NBT ALP staining on days 7 and 14, and matrix mineralization was evaluated by Alizarin Red S staining on days 14 and 21. For osteogenic protein expression, BMSCs cocultured with hydrogels for 7 days under osteogenic induction were collected and lysed in RIPA buffer containing PMSF. Protein concentrations were determined using a BCA assay (Epizyme, CHINA) and equal amounts of protein were resolved by SDS‐PAGE and transferred to PVDF membranes. Membranes were incubated overnight at 4°C with primary antibodies against RUNX2, OPN, OCN, and GAPDH as loading controls. After incubation with HRP‐conjugated secondary antibodies, bands were visualized using ECL and quantified using ImageJ. Simultaneously, confocal microscopy was used to capture immunofluorescence staining results for osteogenic‐related genes.

### 4.9 In Vitro Angiogenesis Assay

HUVECs and BMSCs were cultured under standard conditions and cocultured with the hydrogels under the aforementioned piezoelectric stimulation conditions. For wound healing assays, confluent monolayers were scratched with a pipette tip and imaged at 0, 12, and 24 h, with wound closure quantified by ImageJ. For transwell assays, HUVECs (2 × 10^4^) were seeded in the upper chamber of a transwell insert (8 µm pore size), while hydrogel extract medium with 10% FBS was placed in the lower chamber; migrated cells were fixed, crystal violet‐stained, and counted. For tube formation assays, Matrigel‐coated wells were seeded with HUVECs (1 × 10^5^/well) and incubated with extracts for 12 h, and tubular networks were imaged and quantified using the Angiogenesis Analyzer in ImageJ. For calcein‐AM fluorescence visualization of tube formation, HUVECs were stained with calcein‐AM after incubation, washed with PBS, and imaged under a fluorescence microscope to observe the formed tubular networks.

### 4.10 Intracellular Calcium Imaging

Intracellular Ca^2^^+^ was detected using Fluo‐4 AM staining. Briefly, BMSCs were incubated with Fluo‐4 AM working solution at 37°C according to the manufacturer's instructions and then washed with HBSS to remove excess dye. For endpoint calcium imaging, cells were exposed to hydrogel‐generated piezoelectric stimulation for 30 min, and fluorescence images were acquired immediately after stimulation using a fluorescence microscope. For time‐series calcium imaging, Fluo‐4 AM‐loaded BMSCs were continuously imaged during hydrogel‐generated piezoelectric stimulation to observe dynamic changes in intracellular Ca^2^^+^ fluorescence.

### 4.11 RNA Sequencing and Mechanism Validation

BMSCs were cocultured with ring‐shaped hydrogels under hydrogel‐generated piezoelectric stimulation according to the aforementioned protocol, without direct mechanical loading on the cells. After 7 days of coculture, total RNA was isolated using TRIzol reagent (Takara, Japan). RNA integrity and concentration were assessed prior to library construction, and high‐throughput sequencing was performed by LC‐Bio Technology. Raw reads were processed to remove adapters and low‐quality bases, and clean reads were aligned to the reference genome. Differential gene expression analysis was conducted using DESeq2, with significance thresholds set at *q* < 0.05 and | log_2_ fold change | ≥ 1. The resulting expression profiles were used to generate heatmaps of differentially expressed genes, volcano plots based on q‐values, and Gene Ontology (GO) enrichment analyses. Gene Set Enrichment Analysis (GSEA) was additionally performed to identify coordinated pathway‐level changes associated with piezoelectric stimulation. Key signaling pathways revealed by transcriptomic analysis were subsequently validated at the protein level by Western blot.

### 4.12 Cleavage Under Targets and Tagmentation (CUT&Tag) Assay

Cleavage Under Targets and Tagmentation (CUT&Tag) was performed to profile genome‐wide H3K9ac alterations in BMSCs following piezoelectric (stimulatioSinotech Genomics Co., Ltd, Shanghai). Briefly, ∼1 × 10^5^ BMSCs were collected and immobilized on Concanavalin A–coated magnetic beads, permeabilized, and incubated overnight at 4°C with anti‐H3K9ac antibodies (Abcam, USA) or IgG controls. After secondary antibody binding, the Protein A–Tn5 transposome was added to induce targeted chromatin tagmentation, which was activated by Mg^2^^+^. Fragmented DNA was purified, and sequencing libraries were generated using the VAHTS CUT&Tag Library Prep Kit (Vazyme, China). Paired‐end sequencing was performed on an Illumina NovaSeq platform. Raw reads underwent adapter trimming, quality control, and alignment to the rat reference genome (rn6). Peak calling was conducted using SEACR to identify H3K9ac‐enriched regions. Differential peak analysis was performed with significance thresholds of *p* < 0.05 and |log_2_ fold change| ≥ 0.5, and the results were visualized by genome‐wide heatmaps and volcano plots. Enriched biological processes were further explored using Gene Ontology (GO) functional analysis.

### 4.13 Femoral Condyle Defect Model

All animal procedures followed ARRIVE guidelines and were approved by the Animal Care and Use Committee of Zhongshan Hospital, Fudan University (Shanghai, China) (Ethics approval number: #2024‐041). A total of 48 8 week‐old Sprague–Dawley were randomly divided into four groups: Control, RSF hydrogel, RSF/T‐CMC hydrogel, RSF/T‐CMC/MXene‐CNF piezoelectric hydrogel. At each indicated time point, three rats from each group were used for subsequent experiments. After shaving and disinfection, a longitudinal skin incision of approximately 3 cm was made to expose the femoral condyle. A cylindrical bone defect measuring 3 mm in diameter and 3 mm in depth was carefully created in the femoral condyle using a sterile trephine drill under continuous sterile saline irrigation, without penetrating the opposite cortical bone. Sterilized hydrogels were implanted, and subcutaneous tissue was closed with absorbable sutures. Postoperatively, penicillin (40 mg·kg^−^^1^, Sigma, USA) was administered intramuscularly for 3 days. Calcein (Sigma, USA) and Alizarin Red S (Sigma, USA) were intraperitoneally injected at predetermined time points to label new bone formation. At 6 and 12 weeks, femurs were harvested for micro‐CT analysis (BMD, Tb.N, BV/TV, Tb.Th) and sagittal section observation. Blood biochemistry and histology of major organs at 12 weeks were performed to assess in vivo biocompatibility.

### 4.14 Histological Analysis

At 12 weeks, major organs (heart, liver, spleen, lung, kidney) were collected, fixed in 10% neutral formalin, and subsequently paraffin‐embedded, sectioned, and stained with hematoxylin and eosin (H&E) at Servicebio (Shanghai, CHINA) to assess systemic biocompatibility. For bone samples, femoral condyles were fixed in 4% paraformaldehyde, dehydrated, and embedded in methyl methacrylate. Sections (50–300 µm) were prepared for hard tissue staining with H&E, Toluidine Blue, and Verhoeff–Van Gieson to evaluate bone regeneration and collagen distribution. Immunohistochemistry for H3K9ac was additionally performed to assess epigenetic regulation in new bone tissue.

### 4.15 Calcein/Alizarin Red Double Staining Assay

The mineral apposition rate (MAR) and new bone formation were assessed following established protocols. Briefly, calcein (20 mg kg^−^^1^, Sigma, USA) and alizarin red S (30 mg kg^−^^1^, Sigma, USA) were administered via intraperitoneal injection at 1 and 3 weeks post‐surgery, respectively. At 12 weeks, three rats from each group were sacrificed, and the femoral condyles were harvested, processed for undecalcified sections, and examined under fluorescence microscopy. The mineralization rate was calculated based on the distance between the green (calcein) and red (alizarin) fluorescence labels.

### 4.16 Statistical Analysis

All data are expressed as the mean ± standard deviation (SD). Statistical analyses were performed using SPSS 20.0 software. Differences among groups were assessed by one‐way analysis of variance (ANOVA), followed by post hoc multiple comparison tests where appropriate. A value of *p < 0.05 was considered statistically significant, **p < 0.01 highly significant, and ***p < 0.001 extremely significant.

## Author Contributions

**Yu‐Kai Huang**: conceptualization, methodology, investigation, Writing – original draft, Writing – review and editing, formal analysis. **Juan Li**: conceptualization, methodology, formal analysis, writing – original draft, writing – review and editing. **Miao‐Miao Yang**: conceptualization, methodology, writing – review and editing, writing – original draft. **Chao Jia**: conceptualization, methodology, writing – original draft. **Quan‐Xin Gong**: methodology, formal analysis, writing – original draft. **Guang‐Cheng Yuan**: conceptualization, methodology, formal analysis, writing – original draft. **Dong Zhou**: conceptualization, methodology, formal analysis. **Wen‐Hui Shen**: methodology, conceptualization. **Zeng‐Gan Chen**: methodology, conceptualization. **Zhi‐Rui Dong**: methodology, formal analysis. **Jin‐Wang Huang**: conceptualization, methodology, formal analysis. **Jie Peng**: methodology, formal analysis. **Di‐Han Su**: methodology, formal analysis. **Ming‐Dong Zhao**: conceptualization, supervision, funding acquisition. **Jian Dong**: conceptualization, supervision, funding acquisition. **Li‐Bo Jiang**: conceptualization, supervision, funding acquisition, writing – review and editing.

## Conflicts of Interest

The authors declare no conflicts of interest.

## Supporting information




**Supporting File 1**: advs77911‐sup‐0001‐SuppMat.docx.

**Supporting File 2**: advs77911‐sup‐0002‐VideoS1.avi.

**Supporting File 3**: advs77911‐sup‐0003‐VideoS2.avi.

**Supporting File 4**: advs77911‐sup‐0004‐VideoS3.avi.

**Supporting File 5**: advs77911‐sup‐0005‐VideoS4.avi.

**Supporting File 6**: advs779115‐sup5‐00065‐Video S6.avi.

**Supporting File 7**: advs77911‐sup‐0007‐VideoS6.mp4.

## Data Availability

The data that support the findings of this study are available from the corresponding author upon reasonable request.
