# Supplementary material for: An Injectable and Self‐Reinforced Piezoelectric Hydrogel for Irregular Bone Defects Regeneration: Dynamic Epigenetic Regulation Approach
Source: Adv Sci (Weinh). 2026 Oct 6:e77911. Online ahead of print. doi: 10.1002/advs.77911 (PMC13643114; doi:10.1002/advs.77911)
Supplement: Supplementary file 1 [file ADVS-9999-e77911-s004.docx]

*Supporting Information For*

**Epigenetic programming piezoelectric hydrogel enables regeneration of bone defects**

Yu-Kai Huang^1,2,#^, Juan Li^1,#^, Miao-Miao Yang^3,#^, Chao Jia^1,#^, Quan-Xin Gong^4^, Guang-Cheng Yuan1, Dong Zhou^5^, Wen-Hui Shen^6,7^, Zeng-Gan Chen^1,6,7^, Zhi-Rui Dong ^1^, Jin-Wang Huang^6,7^, Jie Peng^8^, Di-Han Su^1^, Ming-Dong Zhao^2*^, Jian Dong^1,6,7*^, Li-Bo Jiang^1,6,7*^

**July 6, 2026**

*Correspondence:

[jiang.libo@zs-hospital.sh.cn](mailto:jiang.libo@zs-hospital.sh.cn); [dong.jian@zs-hospitcal.sh.cn](mailto:dong.jian@zs-hospitcal.sh.cn); [zhao_mingdong@fudan.edu.cn](mailto:zhao_mingdong@fudan.edu.cn);

**Affiliations:**

^1^ Department of Orthopaedic Surgery, Zhongshan Hospital, Fudan University, Shanghai, 200032, China

^2^ Department of Orthopaedics, Jinshan Hospital, Fudan University, Shanghai, 201508, China

^3^Department of nursing, Zhongshan Hospital Fudan University, Shanghai, 200032, China

^4^ School of Materials and Chemistry, University of Shanghai for Science and Technology, Shanghai 200093, China

^5^ Key Laboratory for Ultrafine Materials of Ministry of Education, Frontiers Science Center for Materiobiology and Dynamic Chemistry, Research Center for Biomedical Materials of Ministry of Education, School of Materials Science and Engineering, East China University of Science and Technology, Shanghai, 200237, P. R. China

^6^ Department of Orthopedic Surgery, Zhongshan Hospital (Xiamen), Fudan University, Fujian, 361015, China

^7^ Xiamen Osteoporosis Specialized Prevention and Treatment Center, Xiamen, 361000, P. R. China

^8^ Clinical Medical College of Xiamen Medical College, Fujian, 361023, China

**Supporting table 1 Primers used for RT-PCR**

| Organism | Gene | Primers |  |
| --- | --- | --- | --- |
| Rat | ALPL | Forward | GACAGACCCTCCCCACTCCT |
|  |  | Reverse | TACCCCGAGATCCGTTCCTC |
| Rat | OPN | Forward | CCAGCCAAGGACCAACTACA |
|  |  | Reverse | AGTGTTTGCTGTAATGCGCC |
| Rat | OCN | Forward | AAGTCCCACACAGCAACTCG |
|  |  | Reverse | TCCATTGTTGAGGTAGCGCC |
| Rat | RUNX2 | Forward | CACAAGTGCGGTGCAAACTT |
|  |  | Reverse | AATGACTCGGTTGGTCTCGG |
| Rat | vWF | Forward | CCGATGCAGCCTTTTCGGA |
|  |  | Reverse | TCCCCAAGATACACGGAGAGG |
| Rat | vEGF | Forward | TTCAAGCCATCCTGTGTGCC |
|  |  | Reverse | CACCAACGTACACGCTCCAG |
| Rat | CD31 | Forward | AACAGTGTTGACATGAAGAGCC |
|  |  | Reverse | TGTAAAACAGCACGTCATCCTT |
| Rat | PTH1R | Forward | GTGGTGGCAGTACCTTGTCC |
|  |  | Reverse | CATGAACTTGAGGCACTCGC |

**Supporting Figures**

**Figure S1.** Dielectric loss (tan δ) curves of PVA, PVA/MXene, and PVA/MXene-CNF composites at room temperature, showing the effect of MXene and CNF incorporation on dielectric dissipation behavior.

**Figure S2.** Photographs showing the comparison between 0.2% MC@TCR and 0.8% MC@TCR hydrogels at room temperature, illustrating their sol-like and gel-like states, respectively.

**Figure S3.** SEM images of 0.8% MC@TCR hydrogel with scale bars of 2 μm and 10 μm.

**Figure S4.** SEM image and corresponding EDS elemental mapping (C, N, O) of the freeze-dried 0.4% MC@TCR hydrogel cross-section at a scale bar of 25 μm.

**Figure S5.** SEM image and EDS elemental mapping (C, N, O, Ti) of the freeze-dried surface of 0.4% MC@TCR hydrogel at a scale bar of 25 μm, including both combined and individual element maps.

**Figure S6.** Cryo-SEM images of RSF, TCR, and 0.4% MC@TCR hydrogels at magnifications of 20,000×, 10,000×, and 5,000×, respectively.

 **Figure S7.** PFM characterization of MXene and MXene-CNF to evaluate their local electromechanical responses. (A) Amplitude–voltage responses of MXene and MXene-CNF. (B) Phase–voltage responses of MXene and MXene-CNF. (C) AFM height, AFM topography, PFM amplitude, and PFM phase images of MXene. (D) AFM height, AFM topography, PFM amplitude, and PFM phase images of MXene-CNF.

**Figure S8.** Additional mechanical characterization of different hydrogels. (A) Representative tensile stress–strain curves of different hydrogels. (B) Normalized stress relaxation curves of different hydrogels under sustained compressive strain.

**Figure S9.** In vitro degradation, swelling behavior, and in vivo functional stability of hydrogels. (A) Remaining weight of RSF, TCR, and 0.4% MC@TCR hydrogels during in vitro degradation in PBS over 28 days. (B) Swelling ratio of RSF, TCR, and 0.4% MC@TCR hydrogels in PBS over 7 days. (C) Compressive stress–strain curves of 0.4% MC@TCR hydrogels harvested after in vivo implantation for 0, 14, and 28 days. (D) Piezoelectric voltage output of 0.4% MC@TCR hydrogels harvested after in vivo implantation for 0, 14, and 28 days.

**Figure S10.** (A) Schematic illustration of the hydrogel piezoelectric measurement system. The hydrogel was sandwiched between copper foil electrodes insulated with Kapton tape and compressed by stainless-steel platens. The generated electrical signals were recorded using a Keithley DMM6500 electrometer. (B) Photographs of the experimental setup showing the compression fixture, electrode connection, and signal acquisition system.

**Figure S11.** Comparison of piezoelectric constants of MC@TCR hydrogels with different MXene:CNF concentration ratios.

**1-Lead screw motor, 2-Control buttons, 3-Display screen, 4-Pressure sensor, 5-Compression plate, 6-Round guide rail, 7-Linear bearing, 8-Spring, 9-Petri dish holder, 10-Base frame**

**Figure S12.** Structural design of the force-sensing and loading-control system of the cyclic compression device.

**Figure S13.** (A) Front, left-side and right-side views of the custom-built hydrogel piezoelectric stimulation device. (B) Photographs showing the ring-shaped hydrogel and its placement in a 35-mm cell culture dish for hydrogel-generated piezoelectric stimulation. (C) Photographs of the custom-built cell force application device equipped with a force-sensing module for monitoring the applied 10 N cyclic compressive force.

**Figure S14.** Schematic and photographic illustration of the in vitro piezoelectric stimulation system. (A) Schematic diagram showing the spatial configuration of the ring-shaped piezoelectric hydrogel and the central cell culture area. The hydrogel was designed to be higher than the culture dish, allowing the compression lid to directly contact and compress the hydrogel during cyclic loading. The generated piezoelectric signals were transmitted to cells through the culture medium. (B) Side-view photograph showing that the ring-shaped hydrogel was higher than the culture dish. (C) Top-view photograph showing the central cell culture area surrounded by the circular piezoelectric hydrogel. (D) Photograph of the cyclic compression device used to mechanically activate the hydrogel.

**Figure S15.** Quantitative analysis of cell viability in control, RSF, TCR, 0.4% MC@TCR, and 0.4% MC@TCR/Com groups after 1, 4, and 7 days of culture, based on Live/Dead staining.

**Figure S16.** Hemolysis assay of RSF, TCR, 0.2% MC@TCR, 0.4% MC@TCR, and 0.8% MC@TCR hydrogels, along with positive and negative controls.

**Figure S17.** Uncropped Western blot images corresponding to the results presented in Figure 6.

**Figure S18.** Calcein-AM fluorescence images of the HUVEC tube formation assay showing capillary-like network formation under different treatments.

**Figure S19.** GSEA plot of the calcium signaling pathway derived from RNA-seq analysis comparing the control and piezoelectric stimulation groups.

**Figure S20.** Representative Fluo-4 AM fluorescence images of BMSCs at 0, 10, 20, and 30 min. Green fluorescence indicates intracellular Ca²⁺ levels.

**Figure S21.** Uncropped Western blot images corresponding to the results presented in Figure 8

**Figure S22.** Uncropped Western blot images corresponding to the results presented in Figure 9.

**Figure S23.** (A) Western blot bands of H3K9ac at 0, 1, 4, 8, 24, and 48 h after hydrogel-generated piezoelectric stimulation. (B) Quantitative analysis of H3K9ac expression normalized to Total H3. ***p < 0.001, **P < 0.01, *P < 0.05, n = 3.

.

**Figure S24.** Uncropped Western blot images corresponding to the results presented in Figure 10.

**Figure S25.** Uncropped Western blot images corresponding to the results presented in Figure 11.

**Figure S26.** Micro-CT three-dimensional reconstruction images of bone defect repair.

**Figure S27.** Blood biochemical indicators of rats in 12 weeks after material implantation.

**Figure S28.** Hematoxylin-eosin staining analysis of different organs in rats sacrificed in 12 weeks after material implantation. Scale bar = 100 µm.

**Figure S29.** (A) Schematic illustration of the piezoelectric measurement setup, in which the hydrogel was implanted into a porcine bone defect and subjected to cyclic mechanical compression. Electrodes were placed on both sides of the hydrogel to record the generated electrical output. (B) Representative photographs of the porcine bone defect model, compression testing setup, and electrical output recording, confirming that the hydrogel retained electromechanical activity under bone-like loading conditions.
